# Fixation probabilities in populations under demographic fluctuations

**DOI:** 10.1007/s00285-018-1251-9

**Published:** 2018-06-07

**Authors:** Peter Czuppon, Arne Traulsen

**Affiliations:** Department of Evolutionary Theory, Max-Planck Institute for Evolutionary Biology, Plön, Germany

**Keywords:** Demographic stochasticity, Fixation probability, Diffusion theory, Evolutionary games, Weak selection, 60J60, 91A22, 92D25

## Abstract

We study the fixation probability of a mutant type when introduced into a resident population. We implement a stochastic competitive Lotka–Volterra model with two types and intra- and interspecific competition. The model further allows for stochastically varying population sizes. The competition coefficients are interpreted in terms of inverse payoffs emerging from an evolutionary game. Since our study focuses on the impact of the competition values, we assume the same net growth rate for both types. In this general framework, we derive a formula for the fixation probability $\varphi$ of the mutant type under weak selection. We find that the most important parameter deciding over the invasion success of the mutant is its death rate due to competition with the resident. Furthermore, we compare our approximation to results obtained by implementing population size changes deterministically in order to explore the parameter regime of validity of our method. Finally, we put our formula in the context of classical evolutionary game theory and observe similarities and differences to the results obtained in that constant population size setting.

## Introduction

The evolutionary dynamics of a mutant strain in a resident population is a well-studied topic in the field of population dynamics. Results concerning the fixation probability, the average fixation time or coexistence behavior can be applied in various biological fields, e.g. population genetics, bacterial evolution, viral dynamics or cancer initiation (Nowak 2006; Altrock et al. 2015; Ewens 2004). While the first theoretical analysis of such processes relied on deterministic differential equations, over the course of time more detailed models were studied describing the stochasticity of microscopic processes on the individual level. These individual based models can be approximated and studied by the replicator equation (in the large population size limit), by branching processes (in case of a small number of invading mutants) or by multi-type birth-death processes (Nowak 2006; Hofbauer and Sigmund 1998; Sandholm 2010; Haccou et al. 2005; Ewens 2004). However, the ecological evolution of the entire population is mostly neglected in these kinds of models and a constant population size is assumed instead. On the other hand, in population genetics and theoretical ecology, studies focused more on the effect that population dynamics have on the fixation probability rather than the concrete interaction mechanisms between the mutant and wild-type individuals (Ewens 1967; Kimura and Ohta 1974; Otto and Whitlock 1997). More recently, researchers started investigating models connecting the stochastic interaction between individuals and stochastic population dynamics from a theoretical point of view (Lambert 2005, 2006; Champagnat and Lambert 2007; Parsons and Quince 2007a, b; Melbinger et al. 2010; Cremer et al. 2011; Gabel et al. 2013; Constable et al. 2016; Chotibut and Nelson 2017). For a historical overview on the calculation of fixation probabilities, see Patwa and Wahl (2008).

To our knowledge, the first analytical approximation of fixation probabilities under stochastically varying population sizes is due to Lambert (2005, 2006). In these papers, the author analyzes models of interacting species by considering the corresponding diffusion equations under the constraint of weak selection. Going one step back on the descriptive scale and analyzing the Kolmogorov forward equation instead of its diffusion approximation, Champagnat and Lambert study the effect of various model parameters on the fixation probabilities and extend the previous results (Champagnat and Lambert 2007). In parallel to these studies, Parsons and Quince examined the effect of variable growth rates on the fixation probability and mean fixation time in a two species system with stochastically varying population size (Parsons and Quince 2007a, b). These results were later complemented and refined in Parsons et al. (2010). Instead of focusing on variable growth rates, in this paper we concentrate on the effect of variable competition coefficients on the fixation probability.

The model we will work with was introduced in Huang et al. (2015). It is a generalized stochastic Lotka–Volterra-model which connects an evolutionary game with the competition coefficients of the model. Individuals reproduce at constant rates and die spontaneously or based on competition within and between species. This leads to stochastically induced demographic fluctuations driven by interactions within the population. We focus on two types only and our goal is to calculate the probability that a mutant takes over such a population of changing size.

Recently, further models have been studied which connected game theoretical dynamics with exogenous population growth (Melbinger et al. 2010; Tao and Cressman 2007; Traulsen et al. 2012; Cremer et al. 2011; McAvoy et al. 2018). For instance, Ashcroft et al. (2017) consider a model with deterministic cell growth defined by a power law and stochastic species interactions derived from an evolutionary game. The authors rely on simulation results suggesting that the evolutionary outcome not only depends on the game played by the species, but also on the growth exponent of the power law governing the population growth.

In the context of public goods dynamics (Constable et al. 2016) study the invasion probability of producers and non-producers of the public good under varying population sizes. Using a time-scale separation under a weak selection approximation, they find that producers can successfully invade a colony of non-producers even though they have a lower fitness than the resident type.

The present paper is structured as follows: In Sect. 2 we describe the generalized Lotka–Volterra-model and restate some basic properties of the system, which were already described in Huang et al. (2015). In Sect. 3 we apply tools developed by Lambert (2006) in order to derive a formula for the fixation probability in the weak selection limit. This allows us to interpret the impact of the competition coefficients separately. Furthermore, we compare the results for various competition matrices induced by different games with each other, i.e. the differences between coordination, coexistence and dominance games. Finally, in Sect. 4 we compare our results to similar models derived in the physics and population genetics literature dealing with deterministic ecological evolution of the model. Furthermore, we examine the fixation probability of a single mutant in a wild-type population, which allows us to compare our findings with those obtained in classical evolutionary game theory, i.e. in finite but fixed population sizes.

## Model

The model we consider is a competitive Lotka–Volterra system consisting of two types, the mutant *X* and the wild-type *Y*. We assume a well-mixed population, i.e. dynamics do not depend on the spatial arrangement of individuals, and a discrete state space describing the number of individuals of the two types, *X* and *Y*.

The evolution of the system is described by birth, death and competition processes, which we assume can be written in terms of chemical reactions. Each individual of the two types can reproduce or die independently of the other individuals. This leads to four reactions for the birth-death-processes,

1 $$\begin{aligned} X \xrightarrow {\beta _X} X + X, \qquad Y\xrightarrow {\beta _Y} Y+Y, \qquad X\xrightarrow {\gamma _X} \varnothing , \qquad Y\xrightarrow {\gamma _Y} \varnothing . \end{aligned}$$

Here, $\beta _X,\gamma _X$ and $\beta _Y,\gamma _Y$ denote the birth and death rates of the mutant and the wild-type, respectively.

Additionally, each individual competes with other individuals and might die due to this process. These reactions occur at the rates

2 $$\begin{aligned} X+X\xrightarrow {\frac{1}{aM}} X,\quad X+Y \xrightarrow {\frac{1}{bM}} Y,\quad X+Y\xrightarrow {\frac{1}{cM}} X,\quad Y+Y \xrightarrow {\frac{1}{dM}} Y, \end{aligned}$$

where *M* controls the total population size in stationarity.

Later on, we interpret the competition rates as inverse payoffs of an evolutionary two-player game with payoff matrixThis interpretation of the competition processes and a descriptive study of the stochastic competitive Lotka–Volterra system as well as a stability analysis of the stationary points of the corresponding deterministic system was performed by Huang et al. (2015). This setup has the advantage that the average size of a monomorphic population reflects the payoffs. For example, focus on a prisoner’s dilemma, where a population of cooperators would be better off than a population of defectors, but selection acts in favor of defectors. A population of cooperators would be larger than a population of defectors, reflecting the fitness values within the population. Letting *X*(*t*) and *Y*(*t*) be the number of mutant and wild-type individuals at time *t*, respectively, the differential equations of the deterministic model that emerges in the large population limit read

3 $$\begin{aligned} \begin{aligned} \frac{dX}{dt}&= X\left( \beta _X-\gamma _X - \frac{X}{aM} - \frac{Y}{bM}\right) ,\\ \frac{dY}{dt}&= Y\left( \beta _Y-\gamma _Y - \frac{X}{cM} - \frac{Y}{dM}\right) . \end{aligned} \end{aligned}$$

For $a>c$ and $d>b$ as well as for $a<c$ and $d<b$, these equations have an internal stationary point where both species exist. It is given by

$$\begin{aligned} (X^*,Y^*) = \left( \frac{ac(b-d)}{bc-ad}(\beta _X-\gamma _X)M,\ \frac{bd(c-a)}{bc-ad}(\beta _Y-\gamma _Y)M\right) . \end{aligned}$$

Its stability depends on whether a coordination ($a>c$ and $b<d$) or a coexistence game ($a<c$ and $b>d$) is played. Additionally, we see that *M* indeed characterizes the scale of the total population size in equilibrium. In the following, we will work with the rescaled variables $x(t)=\frac{X(t)}{M}$ and $y(t)=\frac{Y(t)}{M}$ while the fraction of mutants in the population is given by $p=\frac{x}{x+y}$. We denote the steady state of this value by

$$\begin{aligned} p^* = \frac{x^*}{x^* + y^*}=\frac{ac(b-d)}{ac(b-d)+bd(c-a)}. \end{aligned}$$

Our goal is to extend the analysis of this particular system by approximating the fixation probability of the mutant type *X* in a population of *Y* individuals. The techniques we use rely on the theory of stochastic diffusions, see e.g. Ewens (2004). Hence, we will work with the diffusion approximation of the above system; for a detailed derivation see “Appendix A”. We find the stochastic differential equations

4 $$\begin{aligned} \begin{aligned} x(t)&= x(0) + \int _0^t x(s)\left( (\beta _X-\gamma _X) - \frac{x(s)}{a} - \frac{y(s)}{b} \right) ds \\&\qquad \qquad \qquad + \frac{1}{\sqrt{M}}\int _0^t \sqrt{x(s)\left( \beta _X + \gamma _X + \frac{x(s)}{a} + \frac{y(s)}{b}\right) } dW^1(s),\\ y(t)&= y(0) + \int _0^t y(s)\left( (\beta _Y-\gamma _Y) - \frac{x(s)}{c} - \frac{y(s)}{d}\right) ds \\&\qquad \qquad \qquad + \frac{1}{\sqrt{M}} \int _0^t \sqrt{y(s)\left( \beta _Y + \gamma _Y + \frac{x(s)}{c} + \frac{y(s)}{d}\right) } dW^2(s), \end{aligned} \end{aligned}$$

where $W^1$ and $W^2$ are two independent, one-dimensional Brownian motions. The stochastic integrals are interpreted in the sense of Itô (van Kampen 1997; Gardiner 2004).

The solution of this system of differential equations is a two-dimensional Markov processes with infinitesimal generator given by (see “Appendix A” or Kallenberg 2002, Chapter 21)

5 $$\begin{aligned} Gf(x,y)= & {} x\left( \beta _X-\gamma _X-\frac{x}{a}-\frac{y}{b}\right) \frac{\partial f}{\partial x} \nonumber \\&+ y\left( \beta _Y-\gamma _Y-\frac{x}{c}-\frac{y}{d}\right) \frac{\partial f}{\partial y} \nonumber \\&+ \frac{x}{2 M}\left( \beta _X + \gamma _X +\frac{x}{a}+\frac{y}{b}\right) \frac{\partial ^2 f}{\partial x^2} \nonumber \\&+ \frac{y}{2 M}\left( \beta _Y + \gamma _Y + \frac{x}{c} + \frac{y}{d}\right) \frac{\partial ^2 f}{\partial y^2}. \end{aligned}$$

We now proceed in deriving the fixation probability of the mutant type *X*.

## Fixation probabilities

The main result of this paper is the approximation of the probability of a mutant strain to reach fixation in a resident population of randomly fluctuating size under weak selection. Note first that due to the competition processes neither of the two species is able to go to $\infty$ and hence each of them will die out at a (finite) random time (Lambert 2006). We define fixation of the mutant *X* as follows (see also Lambert 2006, Definition 1.1 for a similar definition):

### Definition 1

(*Fixation*) Species *X* becomes fixed if for some $t\ge 0$ we have $y(t)=0$ and $x(t)>0$.

In order to quantify the fixation probability we make use of the generator description of the model. Let $\varphi (x_0,y_0)$ be the fixation probability of species *X* if the initial type-frequencies are $x_0$ and $y_0$. Then diffusion theory, see also Ewens (2004) and Gardiner (2004) or “Appendix B”, implies that $\varphi$ solves

6 $$\begin{aligned} \left\{ \begin{array}{ll} G\varphi (x_0,y_0) = 0, &{} x_0,y_0 \ge 0, \\ \varphi (x_0,0) = 1, &{} x_0>0, \\ \varphi (0,y_0) = 0, &{} y_0>0. \end{array}\right. \end{aligned}$$

In order to solve this partial differential equation we first do a parameter transformation to the coordinates $p=\frac{x}{x+y}$ and $z=x+y=\frac{X+Y}{M}$, the fraction of *X*-individuals in the population and the whole rescaled population size, respectively. Given the same birth and death rates for both species, i.e. $\beta _X=\beta _Y=\beta$ and $\gamma _X=\gamma _Y=\gamma$, and noting that

$$\begin{aligned} \frac{1}{p^*}= 1+\frac{y^*}{x^*}=1 + \frac{bd}{ac}\cdot \frac{c-a}{b-d}, \end{aligned}$$

the generator transforms to (the detailed calculations are given in “Appendix C”)

7 $$\begin{aligned} \begin{aligned} \widetilde{G}\varphi (p,z)&= \frac{p(1-p)}{d}\left( 1-\frac{d}{b}\right) \left( 1-\frac{p}{p^*}\right) \left( z+\frac{1}{M}\right) \frac{\partial \varphi }{\partial p}\\&\quad + z\left[ \beta -\gamma -\frac{z}{d}\left( 1-p\left( 2-\frac{d}{c}-\frac{d}{b}\right) +\left( 1-\frac{d}{b}\right) \frac{p^2}{p^*}\right) \right] \frac{\partial \varphi }{\partial z}\\&\quad + \frac{p(1-p)}{2zM}\left[ \beta +\gamma + \frac{z}{d}\left( \frac{d}{b} + p\left( 1+\frac{d}{a} - 2\frac{d}{b}\right) - \left( 1-\frac{d}{b}\right) \frac{p^2}{p^*}\right) \right] \frac{\partial ^2 \varphi }{\partial p^2}\\&\quad + \frac{p(1-p)z}{d M} \left( 1-\frac{d}{b}\right) \left( \frac{p}{p^*} - 1\right) \frac{\partial ^2 \varphi }{\partial p \partial z}\\&\quad + \frac{z}{2M}\left[ \beta +\gamma + \frac{z}{d} \left( 1 - p\left( 2- \frac{d}{c} -\frac{d}{b}\right) + \left( 1-\frac{d}{b}\right) \frac{p^2}{p^*} \right) \right] \frac{\partial ^2 \varphi }{\partial z^2}. \end{aligned} \end{aligned}$$

Equation (6) then translates to

8 $$\begin{aligned} \left\{ \begin{array}{ll} \widetilde{G}\varphi (p_0,z_0) = 0, &{} p_0\in [0,1],z_0 \ge 0, \\ \varphi (1,z_0) = 1, &{} z_0> 0, \\ \varphi (0,z_0) = 0, &{} z_0 > 0. \end{array}\right. \end{aligned}$$

From now on, we drop the indices of $p_0$ and $z_0$ since the fixation probability always depends on the corresponding initial values.

Our goal is to approximate the solution of Eq. (8). Therefore, we start with the neutral setting which forms the basis of the subsequent calculations.

### Neutral model

In formal terms, a neutral setting is given when individuals are exchangeable under labelling which in our case is equivalent to choosing a constant competition matrix, i.e. $a=b=c=d$. In this scenario the generator in Eq. (7) simplifies to

$$\begin{aligned} \begin{aligned} \widetilde{G} \varphi _{neu}(p,z)&= z\left( \beta -\gamma -\frac{z}{a}\right) \frac{\partial \varphi _{neu}}{\partial z} + \frac{p(1-p)}{2zM}\left( \beta +\gamma +\frac{z}{a}\right) \frac{\partial ^2 \varphi _{neu}}{\partial p^2}\\&\quad + \frac{z}{2M}\left( \beta +\gamma +\frac{z}{a}\right) \frac{\partial ^2 \varphi _{neu}}{\partial z^2}. \end{aligned} \end{aligned}$$

Solving $\widetilde{G} \varphi _{neu}(p,z) = 0$ with boundary conditions

$$\begin{aligned} \varphi _{neu}(0,z) = 0\quad \text { and }\quad \varphi _{neu}(1,z) = 1 \text { for } z>0 \end{aligned}$$

we obtain $\varphi _{neu}(p,z) = p$, the standard fixation probability of a mutant in an evolutionary process without selection.

### Fixation probability under weak selection

Based on the result of the neutral setting we approximate the fixation probability $\varphi (p,z)$ in the case of weak selection. In our model, this translates to the coefficients of the competition matrix being similar. To be more concrete we need the following conditions

(i) $$(1-\frac{d}{b})^2\ll 1$$,
(ii) $$(1-\frac{d}{b})(2-\frac{d}{c}-\frac{d}{b}) \ll 1$$,
(iii) $$(1-\frac{d}{b})(1+\frac{d}{a}-2\frac{d}{b}) \ll 1$$ and
(iv) $$(1-\frac{d}{b})(\frac{1}{p^*}-2) \ll 1$$.

Condition (i) demands that the parameters *d* and *b* are close together such that their squared difference is negligible. Due to conditions (ii) and (iii) it follows that also *a* and *c* are close to *d* and thus *b* which then implies that the whole competition matrix is just a small perturbation of the neutral matrix in which all entries are the same. Finally, condition (iv) is needed to ensure that there indeed is an internal fixed point in the deterministic system which is close to 1 / 2. For the case that there exists no internal fixed point we refer to Sect. 3.4.

We note again, that we claim that the product of two values which are already small is negligible, the single terms separately however are important for our approximation formula. We see in the derivation of the following theorem that the condition on the equilibrium $p^*$ is necessary for the separation of the variables *p* and *z* reflecting a separation of the ecological and the evolutionary variable. For a extended discussion on the choice of fixed points in these kind of models see also Czuppon and Gokhale (2018).

In the following we will make use of asymptotic notation, i.e.

$$\begin{aligned} \begin{aligned} f(x)&= O(g(x)) \text { for } x\rightarrow 0 \quad&\text { iff } \qquad&\lim _{x\rightarrow 0}\frac{f(x)}{g(x)}<\infty . \end{aligned} \end{aligned}$$

We now state our main result.

#### Theorem 1

(Fixation probability under weak selection) Under conditions (i)–(iv) the solution of Eq. (8) can be written as

9 $$\begin{aligned} \varphi (p,z) = p + p(1-p)\left( 1-\frac{p}{p^*}\right) \left( 1-\frac{d}{b}\right) \psi (z) + O\left( \left( 1-\frac{d}{b}\right) ^2\right) , \end{aligned}$$

where $\psi (z)$ is independent of the initial frequency of mutants *p* and solves

10 $$\begin{aligned} \begin{aligned} 0&= \left( z+\frac{1}{M}\right) + z((\beta -\gamma )d-z) \psi '(z) - \frac{3}{zM}\left( (\beta +\gamma )d+z\right) \psi (z)\\&\quad + \frac{z}{2M}((\beta +\gamma )d+z) \psi ''(z). \end{aligned} \end{aligned}$$

#### Remark 1

We note that this is basically a linearization around the neutral fixation probability and reduces to the neutral model if all payoff coefficients are equal, i.e. $\varphi (p,z)=p$ due to $1-\frac{d}{b}=0$.

The proof of the Theorem is given in “Appendix D”. The idea is to apply the infinitesimal generator $\widetilde{G}$ from Eq. (7) to the formula stated in Eq. (9). Inserting conditions (i)–(iv) then gives the result.

It seems remarkable that the initial population size does not affect the qualitative behaviour of the fixation probability, i.e. $\psi (z)$ does not change signs as we will see later. However, the initial frequency of the mutant compared to the internal steady state and the payoffs *b* and *d* can change the sign of the first order effect under weak selection. An interesting application is to consider fixation out of the neighbourhood of the internal steady state. Precisely at that point, we find $\varphi (p^*,z) = p^*$ under weak selection. This is also implicit in the formulas derived in Lambert (2006), Champagnat and Lambert (2007), and Constable and McKane (2015) but has not been stated explicitly. For $p=p^*+\varepsilon$, we have

11 $$\begin{aligned} \varphi (p^*+\varepsilon ,z) = (p^*+\varepsilon )\left( 1 - \frac{\varepsilon }{p^*}(1-p^*-\varepsilon )\left( 1-\frac{d}{b}\right) \psi (z)\right) + O\left( \left( 1-\frac{d}{b}\right) ^2\right) ,\nonumber \\ \end{aligned}$$

which implies that the fixation probability out of a neighborhood of a stable steady state $p^*$ ($d<b$) is smaller than neutral for positive deviations in *p* and larger than neutral for negative deviations in *p*. On the other hand, for an unstable steady state $p^*$ ($d>b$), the fixation probability out of the neighborhood is larger than neutral for positive deviations in *p* and smaller than neutral for negative deviations in *p*. For a detailed study of fixation probabilities when leaving the deterministic steady state see also Park and Traulsen (2017).

Next, we investigate different competition parameter constellations, i.e. conditions on the evolutionary game. We consider the following cases

- coexistence game    –    $a<c$ and $b>d$;
- coordination game    –    $a>c$ and $b<d$;
- dominance game    –    $a>c, b>d$ or $a<c,b<d$.

The cases (a) and (b) allow for a mixed steady state in the deterministic model given in Eq. (3). For coexistence games, this internal equilibrium is stable whereas for coordination games it is unstable, see for instance (Huang et al. 2015).

A qualitatively different picture arises in case (c). Here, either type *X* or type *Y* strictly dominates the other species in a game theoretic sense. This implies that the deterministic model only allows for single species equilibria where the stationary point of the dominant (inferior) type is stable (unstable). Thus, Theorem 1 does not hold in this case since $p^*$ does not tend to $\frac{1}{2}$. In fact, $p^*$ does not even exist. Instead we will replace condition (iv) by an adapted version which then gives a similar approximation, see Eq. (14) in Sect. 3.4 below.

### Coexistence and coordination games

In this section, we compare the resulting fixation probabilities in a coexistence and coordination game with the neutral fixation probability $\varphi _{neu}(p,z)=p$. In order to do so we need a Lemma characterizing the impact of the initial population size which we prove in “Appendix E”.

#### Lemma 1

The solution $\psi (z)$ of Eq. (10) is positive for all $z>0$.

#### Remark 2

**Fig. 1:**
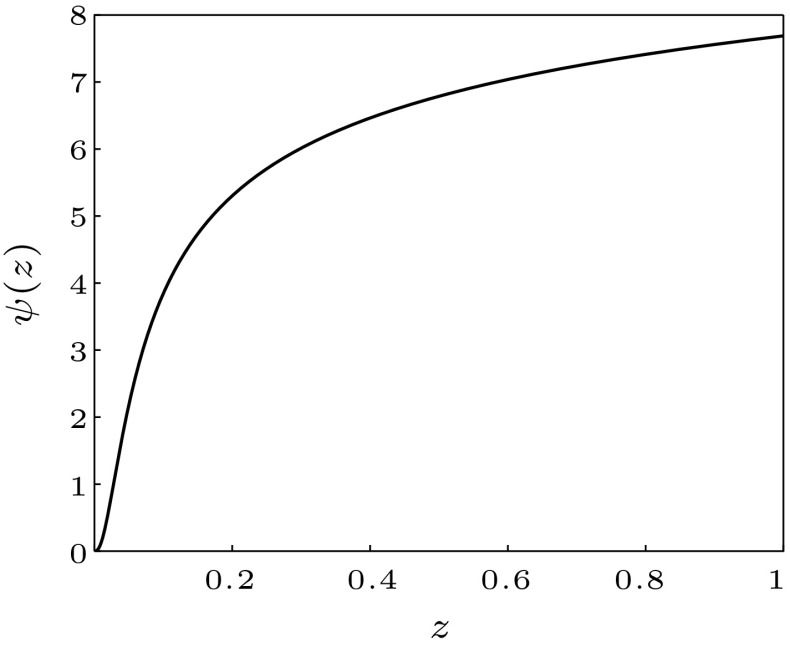
The figure shows the numerical solution of Eq. (10). It remains positive and is growing with increasing *z*. Details on the numerical evaluation of $\psi$ can be found in “Appendix F”. Parameters are given by $d=1, M=100, \beta =0.6, \gamma = 0.1$

In fact the function $\psi$ is a growing function in *z* as can be seen in Fig. 1. This can be explained as follows: since the only difference between the strains are the corresponding competition parameters, regions where these parameters play a dominant role should have a larger impact on the fixation probability. Translating this to initial population sizes yields that larger initial population sizes, being affected mostly by competition processes, also have a larger influence on the evolutionary outcome and thus a larger value for $\psi$.

Now, we can state some immediate consequences of the fixation probability which follow from Eq. (9).

#### Corollary 1

(Impact of competition parameters) Given the assumptions of Theorem 1 we find the following:

1. For arbitrary $a,c>0$ and $p<p^*$ we have that $\varphi >\varphi _{neu}$ iff $b>d$.
2. The probability of fixation is an increasing function in the competition parameter *a*.

#### Proof

Part 1. follows immediately by comparing $\varphi _{neu}$ with $\varphi$ from Eq. (9) and Lemma 1. For part 2., we differentiate the representation of the fixation probability from Eq. (9) with respect to *a* which gives

$$\begin{aligned} \frac{\partial \varphi }{\partial a} = p(1-p)\psi (z) \frac{p}{(p^*)^2} \frac{d c^2(b-d)^2}{(ac(b-d)+bd(c-a))^2} > 0. \end{aligned}$$

The last inequality holds for all choices of the parameter values which finishes the proof. $\square$

#### Remark 3

The first statement of the Corollary has the obvious implication that for a mutant to invade a resident population it is important to perform well against the wild-type. This also implies that species with lower single species equilibria (i.e. $a<d$) can have a higher chance of fixating than neutral. This can end up in a decrease of the overall population size. But still, as the second part of the Corollary shows, species with higher single-species equilibria also have a higher chance to reach fixation.

Before turning to dominance games we take a brief look at some special cases in the context of coexistence and coordination games.

#### Symmetric and asymmetric games

Here, we assume that $a=d$ and distinguish between symmetric games, i.e. $b=c$ and asymmetric games, $b\ne c$. In the case of symmetric games we have $\frac{a}{c}=\frac{d}{b}$ and thus $p^* = 1/2$. Therefore the fixation probability in Eq. (9) simplifies to

12 $$\begin{aligned} \varphi _{sym}(p,z) \approx p + p(1-p)(1-2p)\left( 1-\frac{d}{b}\right) \psi (z). \end{aligned}$$

**Fig. 2:**
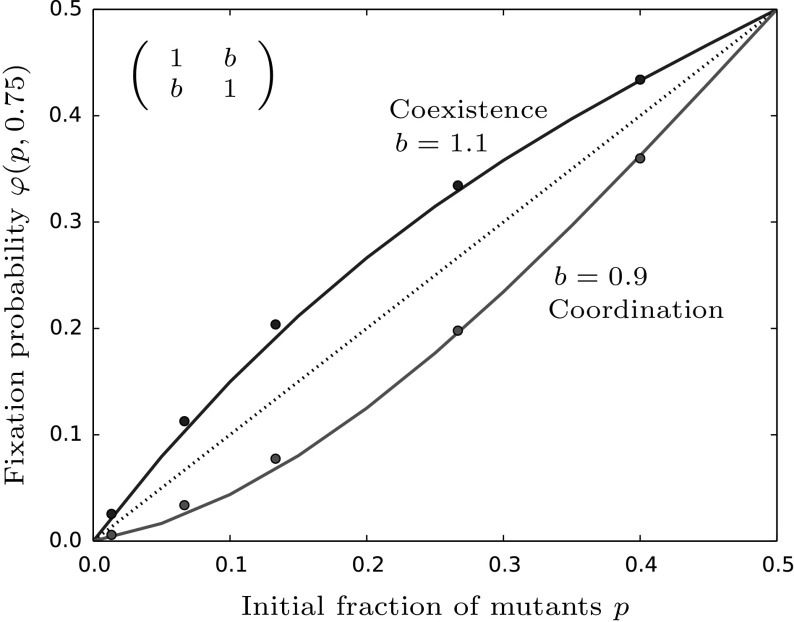
The figure shows the fixation probability from Eq. (12) compared to the neutral fixation probability given by the dashed line. For coexistence games it is higher whereas in coordination games it is lower than the neutral values. The parameters are given by $a=d=1,b=c=0.9/1.1,M=100,\beta =0.6,\gamma =0.1$ and $z=0.75$. The bullets are averages taken over 100, 000 simulations. For the numerical solution of $\psi (z)$, see “Appendix F”

Additionally, note that in this case we do not need assumption (iv) for the solution of the generator equation in (8). For an illustration of the fixation probability with some simulated data points, see Fig. 2. For coexistence games ($b>d$) the fixation probability lies above the neutral line while for coordination games ($b<d$) the fixation probability is lower. Choosing *b* closer to *d* improves the analytical prediction due to the weak selection approximation in conditions (i)–(iii).

For asymmetric games, i.e. we still assume $a=d$ but now $b\ne c$, we obtain similar results. In this case, the fixation probability is given by

13 $$\begin{aligned} \varphi _{asym}(p,z) \approx p + p(1-p)\left( 1-\frac{p}{p^*}\right) \left( 1-\frac{d}{b}\right) \psi (z). \end{aligned}$$

**Fig. 3:**
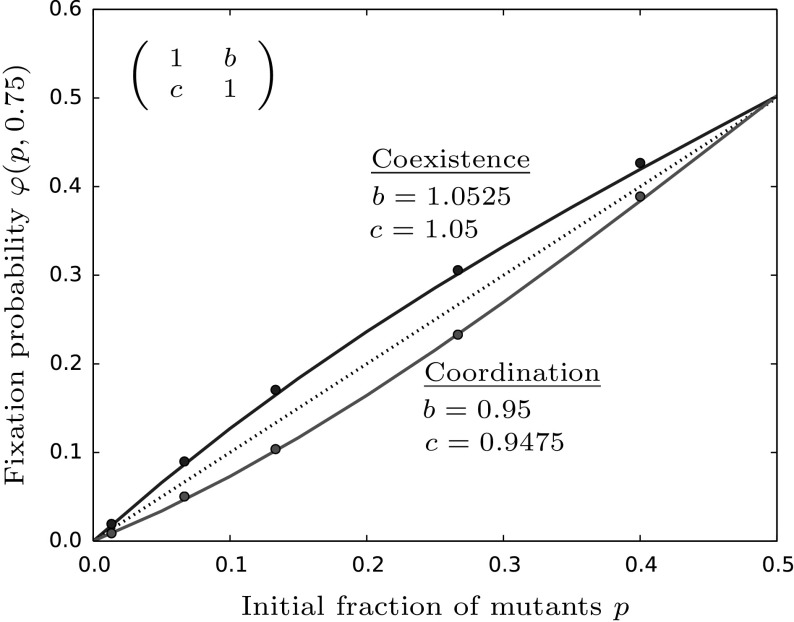
The fixation probability under asymmetric competition coefficients from Eq. (13) is compared to the neutral fixation probability displayed by the dashed line. As in the symmetric case, coexistence games give higher and coordination games give lower values than the neutral model, respectively. Parameters are chosen as follows: $a=d=1,M=100,\beta =0.6,\gamma =0.1,z=0.75$ and *b*, *c* as given in the figure. The points are averages taken over 100,000 stochastic simulations of the original model and $\psi (z)$ is evaluated according to “Appendix F”

Dependent on whether $\frac{d}{b}>1$ (coordination) or $\frac{d}{b}<1$ (coexistence) the resulting fixation probability again lies below or above the neutral value, respectively, see also Fig. 3.

The condition for invasion, i.e. for $\varphi _{asym}>\varphi _{neu}$ to hold, is again given by $b>d$. Thus, for a mutant to reach fixation in the resident population, it is primarily important to have a high payoff when playing against the resident, i.e. the more abundant type. However, when comparing $\varphi _{sym}$ and $\varphi _{asym}$ we see that here the parameter *c* does play a role. To be more precise, whenever $b>c$ we have $\varphi _{asym}>\varphi _{sym}$. This condition resembles the shifting of the internal equilibrium of the deterministic system towards the mutant-axis, i.e.

$$\begin{aligned} p^*_{asym}> p^*_{sym}=\frac{1}{2} \qquad \text {iff} \qquad b>c. \end{aligned}$$

### Dominance game

**Fig. 4:**
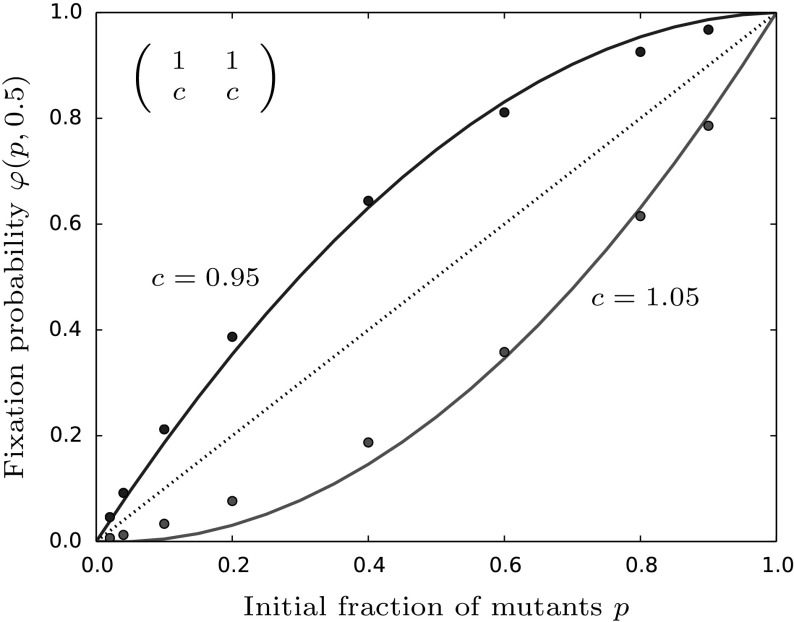
The fixation probability in the case of one strain dominating the other. The plot shows the theoretical prediction taken from Eq. (14) together with the neutral fixation probability displayed by the dashed line. In contrast to the previously studied parameter configurations we do not have internal fixed points of the deterministic system such that the solid lines remain above or below the diagonal. Parameters are chosen as follows: $a=b=1,M=100,\beta =0.6,\gamma =0.1,z=0.5$ and *c* as given in the figure. The points are averages taken over 100,000 stochastic simulations of the original model and $\psi (z)$ is evaluated according to “Appendix F”

In contrast to coexistence and coordination games the dominance game does not have an internal equilibrium in its deterministic counterpart. This is due to one strategy strictly dominating the other. In terms of parameters this means that either $a>c$ and $b>d$ or $a<c$ and $b<d$ hold. As already mentioned, for the analysis of the fixation probability it does not make sense to assume condition (iv) which states that the internal equilibrium should be close to $\frac{1}{2}$. Hence, we can already infer that the analytical solution will not intersect with the neutral line due to one strategy being favored independently of its frequency (Fig. 4).

For the calculation of $\varphi _{dom}$ we still assume conditions (i)–(iii) but instead of condition (iv) we need the following:

(v) $$\left( 1-\frac{d}{b}\right) \left( 1+\frac{db}{ac}\frac{c-a}{b-d}\right) \ll 1$$.

This is plausible since the fraction in the last term should approximate $-1$ when considering a dominance game under weak selection, i.e. either $c>a$ and $d>b$ or $c<a$ and $d<b$.

#### Theorem 2

Under conditions (i)–(iii) and (v), we find

14 $$\begin{aligned} \varphi _{dom}(p,z) = p + p(1-p)\left( 1-\frac{d}{b}\right) \psi (z) + O\left( \left( 1-\frac{d}{b}\right) ^2\right) , \end{aligned}$$

where $$\psi (z)$$ satisfies

15 $$\begin{aligned} 0= & {} \frac{1}{d}\left( z+\frac{1}{M}\right) + z\left( \beta -\gamma -\frac{z}{d}\right) \psi '(z)-\frac{1}{zM}\left( \beta +\gamma +\frac{z}{d}\right) \psi (z) \nonumber \\&+\, \frac{z}{2M}\left( \beta +\gamma +\frac{z}{d} \right) \psi ''(z). \end{aligned}$$

The proof is an imitation of the proof of Theorem 1 and therefore spared out.

We see that indeed $\varphi _{dom}$ is always larger or smaller than $\varphi _{neu}$ dependent on *b* being larger or smaller than *d*, respectively. This finding is rather trivial, since we consider a dominance game and $b>d$ ensures the mutant being advantageous. More importantly, Eq. (14) allows to calculate the first order approximation of the neutral result.

## Discussion

Now that we have derived the fixation probabilities in all possible scenarios captured by our model, we can compare them to previously derived results. Since our model takes into account stochastic fluctuations in population size, it is especially interesting to see how that affects the fixation probability in comparison to deterministically varying or constant population sizes. Concerning constant population sizes we also compare our results to the $\frac{1}{3}$-rule which deals with the invasion of a single mutant into a wild-type population.

### Comparison to models with deterministically varying population size

Fixation probabilities under deterministically changing population sizes have been extensively studied in the population genetics literature (Uecker and Hermisson 2011; Otto and Whitlock 1997; Kimura and Ohta 1974; Waxman 2011; Wahl and Gerrish 2001). It is possible to, at least partially, compare our results to these models. In order to do so, we consider the case of a dominating trait with frequency independent selection, i.e. we are in the context of Theorem 2 with parameters being equal along the rows of the competition matrix, i.e. $a=b$ and $c=d$. This transforms the competition being only dependent on the population density and not on the population composition. Then typically $s=a-c$ represents the fitness advantage of a mutant when compared to a wild-type resulting in a higher replacement (or reproduction) rate of wild-type individuals. This effect cannot easily be translated to our mechanistic model in (1) and (2). In our model selection happens due to competition and thus affects the death rates of individuals, see (Baar et al. 2017) for a similar model in the adaptive dynamics framework. To account for that we should set $s=\frac{1}{d}-\frac{1}{b}$ which is already part of the first order term in our derived approximations in equations (9) and (11), see also Lambert (2006) for a similar definition of selection in a similar model.

Using the selection coefficient *s* and the population size *M*, a classical assumption in population genetics is $Ms\gg 1$ which is violated in our setting since our approximation is applicable when $Ms<1$ which emerges from the selection interaction modeled by the microscopic competition reactions stated in (2). Furthermore, once an individual dies due to a competition event it does not get replaced by another one, allowing for fluctuating population sizes. This is, as far as we can judge, different to models with varying population size in existing population genetics literature where the population size dynamics are often separated from the evolutionary dynamics. These two differences prevent a rigorous comparison between results obtained in the population genetics context and our microscopic model.

Employing a mechanistic individual based model as we do has both advantages and disadvantages. As a drawback, one could argue that externally driven fluctuations in population size, for example seasonal fluctuations or bottleneck experiments as analyzed in Uecker and Hermisson (2011), and Wahl and Gerrish (2001), cannot be dealt with—at least not in our studied model. On the other hand the microscopic model allows for concrete interpretation of the selection dynamics and allows us to explore the stochastic effects in small populations in more detail. That is, as opposed to a general selection value *s* affecting the birth rate, we can study the specific impact of varying the different parameters corresponding to single reactions much like it was performed in Lambert (2006), Champagnat and Lambert (2007), Parsons and Quince (2007a, b), Parsons et al. (2010), and Czuppon and Gokhale (2018). This microscopic approach has recently received some attention, see Doebeli et al. (2017).

We now proceed to compare our approximation with those obtained in the literature considering similar scenarios.

#### Deterministic logistic growth—Otto and Whitlock (1997), Kimura and Ohta (1974)

In Otto and Whitlock (1997) the authors derive a stochastic diffusion approximation for populations consisting of two types, one strictly dominant, under frequency-independent selection and deterministically varying population sizes. In case of logistic growth they find in their Eq. (11) for the fixation probability of a single mutant

16 $$\begin{aligned} \varphi _{OW}\left( \frac{1}{zM}\right) = \frac{2sK(s+r)}{sK + rzM}, \end{aligned}$$

where *s* is the selection advantage, *K* the carrying capacity and *r* the logistic growth parameter. These parameters can be translated to our setting. As the selection strength we set $s=\frac{1}{d}-\frac{1}{b}$ which is the selective advantage of the wild-type in the frequency-independent case, i.e. $a=b$ and $c=d$. The carrying capacity is set to $K=(\beta -\gamma )aM$ and $r=\beta -\gamma$ is the effective growth.

**Fig. 5:**
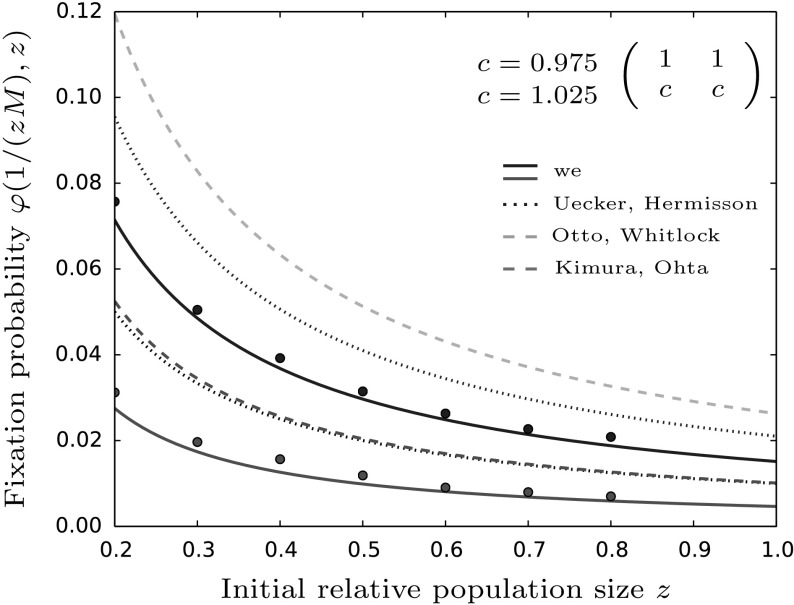
We compare predictions for fixation probabilities of a single mutant in a frequency-independent dominance scenario. The solid lines are our result from Theorem 2 while the dotted line corresponds to the approximation derived in Uecker and Hermisson (2011) and the dashed lines are results from Otto and Whitlock (1997) (orange) and Kimura and Ohta (1974) (green). All these results correspond to the case $c=0.975$. We see that our approximation fits the simulation results best which is mainly due to the assumption $Ms\gg 1$ being violated in our framework but necessary for the derivations in the other references. Parameter values are: $a=b=1,M=100,p=\frac{1}{zM},\beta =0.6,\gamma =0.1$ and $c=d$ as given in the figure. For the translation of parameters between the models see Sects. 4.1.1 and 4.1.2 (color figure online)

The equation derived by Otto and Whitlock is valid for $s,r< 0.1$ and large population sizes *zM*. Since neither $r<0.1$ nor $zM\gg 1$ are met in our simulations it is not surprising to see deviations between our approximation and (16), see also Fig. 5.

Further, the authors mention that when the conditions $s,r<0.1$ do not hold, one can use the approximation derived by Kimura and Ohta (1974). There, the Kolmogorov backward equation is used and the result they obtain is given by (see equation (5) in Kimura and Ohta 1974 or (A1) in Otto and Whitlock 1997)

17 $$\begin{aligned} \varphi _{KO}\left( \frac{1}{zM}\right) = \frac{1-\exp \left( -4 Q s \frac{1}{zM}\right) }{1-\exp (-4 Q s)}, \end{aligned}$$

where *Q* is given by (see Eq. (A8) in Otto and Whitlock 1997)

$$\begin{aligned} Q = \frac{KzM(s+r)}{sK+rzM}. \end{aligned}$$

However, for the derivation of the equations to hold, one needs $Qs\gg 1$ which is not satisfied in our setting such that again it is not unexpected to see differences between the two approximations, see Fig. 5.

Due to the underlying assumptions, these approximations only work in case of an advantageous mutant, i.e. $s>0$. Therefore, in the figures we only compute the corresponding values in case of $a=b>c=d$.

#### Deterministic logistic growth—Uecker and Hermisson (2011)

The model developed by Uecker and Hermisson (2011) is different to the previous one since it strictly distinguishes between ecological and evolutionary steps. On the ecological scale both types are the same while due to evolutionary updating the fitter type on average replaces the other type more often. Since selective advantage is again frequency-independent we are again in the case of dominance. Their result for logistic growth and constant selective advantage, equation (24) in Uecker and Hermisson (2011) reads

18 $$\begin{aligned} \varphi _{UH}\left( \frac{1}{zM}\right) = \frac{s(r+s)}{s(r+s)+(b+\xi )(s+rzM/K) + rzM(r+s)/K}, \end{aligned}$$

where *s* is the selective advantage of the mutant, *r* the logistic growth rate, *b* the birth rate of individuals, $\xi$ the neutral evolutionary replacement rate and *K* the carrying capacity. Translating our modeling parameters into this setting we obtain $s=\frac{1}{d}-\frac{1}{b}$, $r=\beta -\gamma$, $b=\beta$, $\xi =0$ and $K=raM$. We note, that the choice of $\xi$ is to some extent arbitrary. We chose to set it to 0 since we do not have any comparable mechanism in our model.

The authors note that for $\varphi _{UH}(1/(zM)) M \gtrsim 10$ the approximation in Eq. (18) fits excellently to the simulation results. However, again this does not belong to the range covered by our approximation such that we find a disagreement between the approximations, see Fig. 5.

In contrast to the derived approximations before, we can use the method from Uecker and Hermisson (2011) to solve for varying initial frequencies. Even though the branching process assumption only makes sense for an invading and initially rare mutant, the formula can be applied to larger values of *p*. Their equation (14) together with (24) then give

19 $$\begin{aligned} \varphi _{UH}(p,z) = 1-\left( 1- \frac{s(r+s)}{s(r+s)+(b+\xi )(s+rzM/K) + rzM(r+s)/K}\right) ^{pzM}. \end{aligned}$$

A visual comparison of this result with our approximation is given in Fig. 8. As we see there, the branching process approach overestimates the simulation results. This discrepancy can again be attributed to $\varphi _{UH}(1/(zM))M \gtrsim 10$ not being satisfied.

However, the branching process approach is, as the approximations by Otto and Whitlock and Kimura and Ohta, limited to the case of beneficial mutations, i.e. $s>0$. We therefore compute only the case $a=b>c=d$ for this approximation in the figures below.

##### Remark 4

It might seem odd that both branching process estimates obtained by Otto/Whitlock and Uecker/Hermisson overestimate the actual fixation probability. Usually, branching process approaches tend to underestimate fixation probabilities since they do not account for the absorbing state at $p=1$. However, the overestimation in these cases comes from the distinct implementation of the selection processes. While in our model selection acts implicitly through the competition processes, in the mentioned papers it is explicitly added to the birth rate of the mutant. The complicated intertwining of selection and population ecology in our model causes difficulties in computing the fixation probabilities in the same vein as suggested in the branching process approaches. On the other hand the added selective advantage to the birth rate causes the mutant to have a higher reproductive rate independent of the population composition and size which does not hold either in our case. Hence the overestimation of the results obtained in Otto and Whitlock (1997) and Uecker and Hermisson (2011).

#### Nearly constant population size—Constable and McKane (2015)

We now want to compare our results to a model with nearly constant population size, i.e. basically constant population size with small fluctuations around the ecological equilibrium. Therefore, we use a time-scale separation argument, developed by Constable and McKane (2015) in the case of Lotka–Volterra dynamics.

**Fig. 6:**
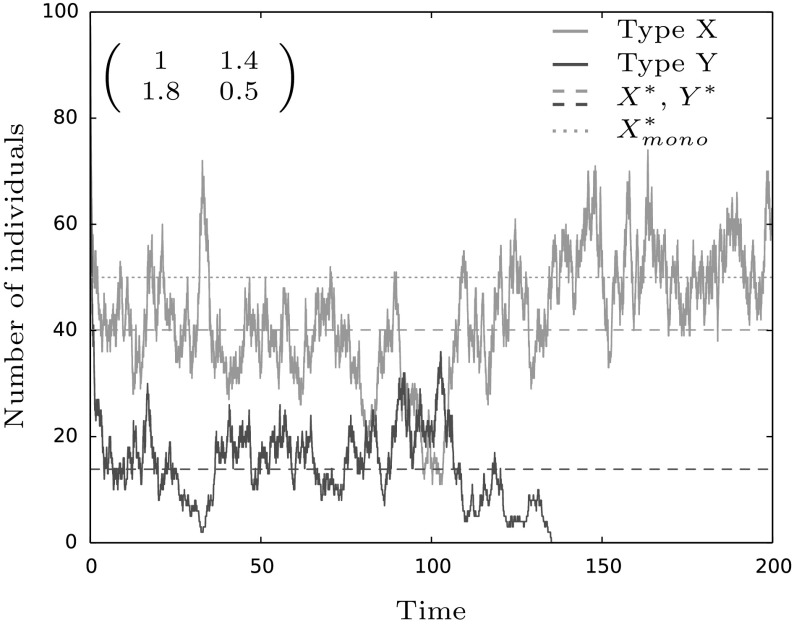
A stochastic simulation of the dynamics in a coexistence game. One can see that the trajectories quickly enter the neighborhood of the deterministic equilibria of the system, displayed by the dashed lines. Further, once type *Y* goes extinct, the blue trajectory approximates the monomorphic equilibrium of *X* visualized by the blue dotted line. This shows that a time-scale separation is indeed a reasonable assumption in a system under Lotka–Volterra dynamics. The initial condition is $X_0=100$ and $Y_0=100$. Competition parameters are given by the matrix in the figure and remaining parameters are chosen as: $\beta =0.6,\gamma =0.1$ and $M=100$

In order to do so, we distinguish between the fast ecological scale and the slow evolutionary process. This is reasonable since due to our choice of reaction rates in Eqs. (1) and (2) we see that the population quickly equilibrates. More precisely, looking at Eq. (3) we see that for a initial population size of order *O*(1) the process first grows until it reaches size *O*(*M*) since until then the competition terms, which are of order $O(M^{-1})$, can be neglected. A similar reasoning shows that for initial population sizes of order $O(M^\alpha )$ for $\alpha >1$ the population decreases to order *O*(*M*) since the linear birth and death terms are negligible, at least when compared to the competition rates. Thus, competition is the key driver in this case. This reasoning is true as long as *M* is sufficiently large such that the birth-death and competition processes can be separated. As we see in Fig. 6, choosing $M=100$ is already large enough to observe this time-scale separation.

For the formal approximation of the population equilibrium we use the projection technique developed in Constable and McKane (2015, 2017) which maps the two-dimensional dynamics onto a one-dimensional manifold. Briefly, the method works in the context of weak selection, i.e. in our case small differences of the competition parameters. Additionally, the neutral model needs to exhibit a stable center manifold which holds for general Lotka–Volterra dynamics. Under these assumptions one ends up with a stochastic diffusion along this manifold which can then be analyzed by stochastic diffusion techniques, i.e. making use of the scale function of the diffusion process (for details see Ewens 2004, Chapter 4.3). Translating the results obtained in Constable and McKane (2015) to our model we obtain the following diffusion (the derivation is given in “Appendix G”—see also equation (14) in Constable and McKane (2015))

20 $$\begin{aligned} dq = q(1-q)\left( \frac{1}{d}-\frac{1}{b}-\left( \frac{1}{a}+\frac{1}{d}-\frac{1}{b}-\frac{1}{c}\right) q\right) dt + \sqrt{\frac{2 \beta q(1-q)}{M(\beta -\gamma )^2}}dW_t, \end{aligned}$$

where $q=\frac{x}{a(\beta -\gamma )}$ is the variable along the center manifold of constant population size.

**Fig. 7:**
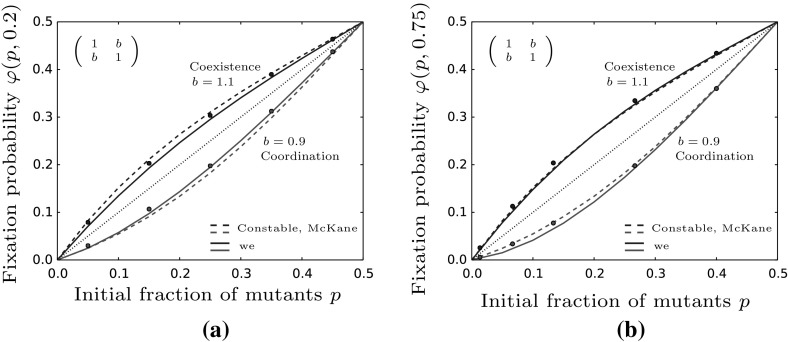
We compare the prediction using a average population size, Eq. (20), (dashed colored lines) and our approximation (solid lines) in the case of bistable and coexistence dynamics. In **a** the initial population size is set to 20 while in **b** it is 75. The figures show that both predictions give very similar values and also produce similar accuracies to the simulation results. Parameters are chosen as stated in the figures with $M=100$, $\beta =0.6$, $\gamma =0.1$ and $a=d=1$. Bullets are averages computed from 100,000 simulations

We now compare our results for the fixation probability with those obtained by using (20). As we can see in Fig. 7 the two results are very similar in the cases of coexistence and coordination game dynamics. Looking at the subfigures of Fig. 7, we only observe small differences between the two approaches. The largest deviation between the two models arises in the bistable case in subfigure (a) where our result is slightly more accurate. In that case we consider initial population sizes smaller ($z=0.2$) than the carrying capacity on the center manifold ($z=0.5$). Interestingly this effect is reversed when starting above the center manifold, see subfigure (b). A possible explanation might be an overestimation of stochastic fluctuations by our approach in (b) and an underestimation of stochastic effects by Eq. (20) in (a). The overestimation of stochasticity would also explain our approximation often being smaller than the observed fixation probabilities for small initial mutant populations ($\sim p$ smaller than 0.3). Fluctuations can drive the rare mutant type to extinction while deterministic dynamics pull it to the center manifold where the reasoning derived in Constable and McKane (2015), our Eq. (20), is valid. If deterministic dynamics are strong enough (or stochasticity weak enough) initial fluctuations can be neglected which corresponds to the approach taken in Constable and McKane (2015).

**Fig. 8:**
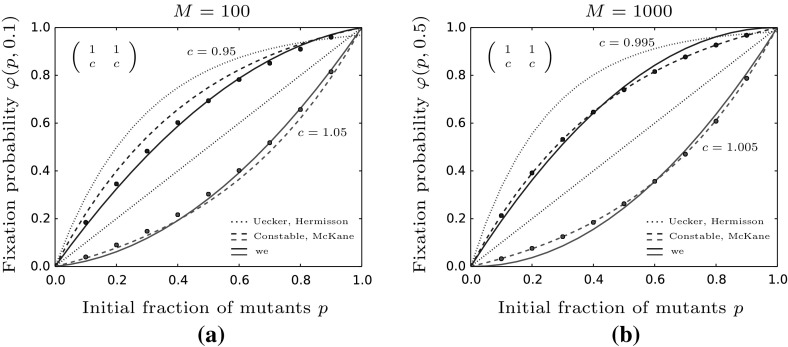
We plot the fixation probability of a dominance game for **a** $M=100$ and **b** $M=1000$. **a** For smaller population sizes and initial values far below the center manifold our approximation from Theorem 2 (solid lines) is more accurate while **b** for larger population sizes the predictions obtained in Constable and McKane (2015) (colored dashed lines) fit the simulation results excellently. Additionally, we plot the result derived in Uecker and Hermisson (2011) (dotted line) which overestimate the fixation probability in both cases. Parameters are chosen as follows: $a=b=1$, $\beta =0.6$, $\gamma =0.1$ and $c=d$ as stated in the figure. Dots represent simulated fixation probabilities obtained from 100,000 simulations (color figure online)

In case of a dominant strain, we observe some differences. As we can see in Fig. 8a our result captures the simulation results slightly better than the approximation from Constable and McKane (2015). This difference emerges since we do not start close to the center manifold but at $z=0.1$. Hence, the initial value on the center manifold does not agree or is not close enough to the initial frequency of mutants of the simulations. For techniques of how to estimate the initial value on the slow sub-system in a more sophisticated way we refer to Constable and McKane (2018) and Roberts (1989). This difference however vanishes when we increase the population size as done in subfigure (b) which also means that we decrease the impact of stochastic fluctuations. This can be seen by our result being slightly off the data points whereas the model derived in Constable and McKane (2015) neglecting the demographic fluctuations closely follows the simulation results.

Summarizing the comparison between the different models we observe that for lower population sizes ($M\sim 100$) where stochastic fluctuations have a strong impact on the model dynamics, our approximation which explicitly accounts for these fluctuations, provides the best fit. For intermediate population sizes ($M\sim 1000$) our approximation seems to overestimate the effect of demographic stochasticity and thus methods like the projection of Constable and McKane describe the evolutionary trajectories best. However both, our approximation as well as the time-scale separation work with very weak selection strengths ($s=1-\frac{d}{b}$) such that $Ms=O(1)$. Other scalings of *Ms* yield different limiting models such that other techniques are necessary for analyzing the fixation probability. Therefore, e.g. Uecker and Hermisson use branching processes to study a system with a larger population size (or selection strength) shifting *Ms* to larger values. Their results work best for parameter choices which yield $Ms>10$ and provide better fits than the previously mentioned approximations.

##### Remark 5

In Fig. 8b we chose $M=1,000$ which is in population genetics terms still a small population. Unfortunately, for values of *M* larger than that we ran into numerical problems (unstable convergence of the method) solving the differential Eq. (15) for $\psi$.

### Fixation of a single mutant under frequency-dependent selection

**Fig. 9:**
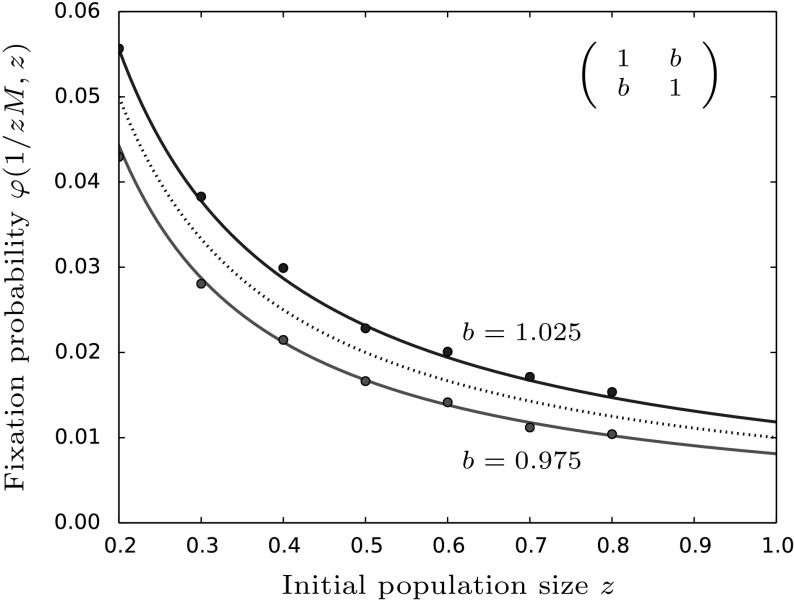
The fixation probability of a single mutant, Eq. (12), under varying initial population sizes is shown in the case of a coexistence and coordination game. Again, these are separated by the neutral fixation probability (dashed line). The model parameters are set to $a=d=1,M=100,\beta =0.6,\gamma =0.1$ and $b=c$ as stated in the figure. The red line refers to the case of coordination or bistability in which context the classical $\frac{1}{3}$-rule was derived. The points are averages taken over 100, 000 simulations

In this section, we consider the case that $p=\frac{1}{zM}=\frac{1}{1+Y_0}$, i.e. initially there is exactly one mutant present in the population. This is probably the most interesting scenario as seen from a biologist’s perspective. In contrast to the previous sections we fix the initial fraction of mutants and instead vary the initial population size *zM*. As can be seen in Fig. 9, the probability of fixation is a decreasing function of the initial population sizes. This translates to the already observed fact that fixation of a mutant strain is more likely in a smaller population and therefore initially growing population than in a larger one which is initially decreasing, cf. Kimura and Ohta (1974). We can also infer this from the formula describing the fixation probability: since we are working in the weak selection limit the governing part of $\varphi (p,z)$ is the initial frequency of mutants, here $\frac{1}{zM}$, which is decreasing for increasing *z* which means that $\varphi$ is a decreasing function in *z*.

#### Comparison to models with constant population sizes

In models with a constant population size *N*, the fixation probability of a mutant strain under weak selection can be translated to the location of the mixed equilibrium of the corresponding replicator equation. More specifically, in the case of bistability (coordination game) a mutant has a higher probability than neutral, i.e. $\frac{1}{N}$, to invade a resident population if the basin of attraction of the wild-type is smaller than $\frac{1}{3}$. This is referred to as the $\frac{1}{3}$-rule first derived in Nowak et al. (2004) and later generalized in scope in Lessard and Ladret (2007); Pfaffelhuber and Wakolbinger (2018). An equivalent approximation has also been made for multiplayer games (Kurokawa and Ihara 2009; Gokhale and Traulsen 2010; Lessard 2011). However, as noted in Traulsen et al. (2006) and Sample and Allen (2017) the order of limits is relevant for the rule to hold. One first needs to take the weak selection limit and then perform an approximation for large population sizes *N*.

Just by looking at our conditions for the approximation to hold, especially condition (iv) stating that $p^*\approx \frac{1}{2}$, we would not expect the $\frac{1}{3}$-law to hold. Still, we are able to draw some more general conclusions from our approximation and those obtained in Constable and McKane (2015) which suggests that a similar rule may not hold in populations with fluctuating size.

**Fig. 10:**
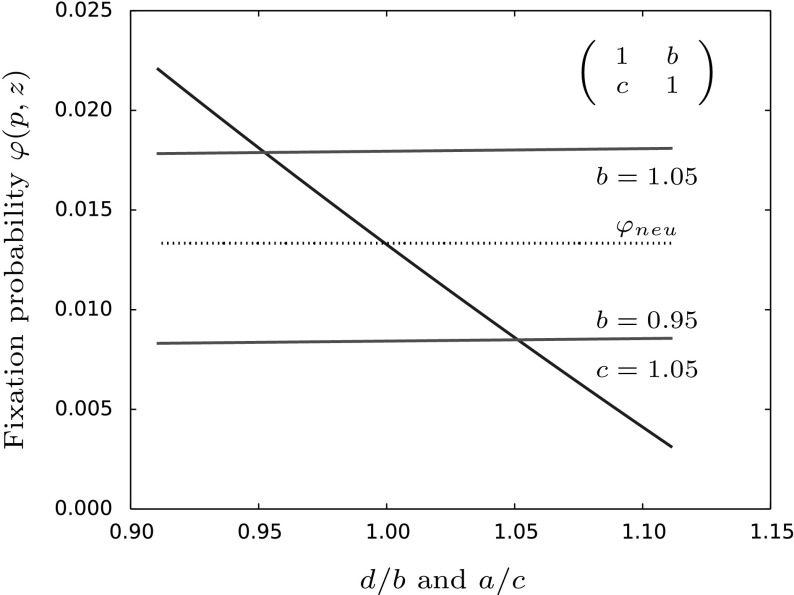
This figure shows the impact of the competition parameters on the fixation probability given in Eq. (9). Fixing *a*, *b* and *d* and varying *c* the fixation probability stays above or below the neutral fixation probability dependent on the choice of *b*. This is not true when we vary *b* but set *a*, *c* and *d* to a constant value. This illustrates that indeed the evolutionary chance for a mutant to become fixed is qualitatively independent of *c* and completely determined by *b* and *d*. Parameter values are: $a=d=1,z=0.75,M=100,p=\frac{1}{zM},\beta =0.6,\gamma =0.1$ and *b*, *c* as given in the figure

Our analytical approach relies on a stochastic diffusion equation which depends on a large population size approximation, i.e. $N\gg 1$ or in our case $M\gg 1$. Just after that approximation we let selection be weak which translates to the competition coefficients being similar. Thus again, it should not be surprising that the $\frac{1}{3}$-rule is violated in our model. Instead, the invasion probability only depends on the competition rates *b* and *d* describing the competition pressure of the species due to the resident type. However, these do not imply any properties of equilibria in the corresponding deterministic system. Even more, the choice of the parameters *a* and *c* does not affect the qualitative behavior of the fixation probability when compared to the neutral case as was already pointed out in Corollary 1. Thus, in the present model the difference between the neutral fixation probability and the probability of fixation under weak selection can be entirely described by the parameter $\frac{d}{b}$ and is independent of $\frac{a}{c}$ as can be seen in Fig. 10.

Still, our approximation of the fixation probability is in some sense similar to the approximation obtained by first taking the weak selection limit and then the population size approximation. In Nowak et al. (2004), and Lessard and Ladret (2007) the fixation probability takes the form

$$\begin{aligned} \varphi _{\text {constant pop size}} \approx \frac{1}{N} + O(\omega ,p^*), \end{aligned}$$

where $\omega$ is the selection intensity. This has the same form as our result obtained in Theorem 1 with $\omega = 1-\frac{d}{b}$. The reason for this is that even though we perform a diffusion approximation, we still let *N* (or rather *M*) be finite which results in the first term $\frac{1}{zM}$ being non-zero in contrast to being 0 for $M\rightarrow \infty$. Additionally, our first order term also involves the deterministic equilibrium $p^*$ which is again reminiscent of the formulas derived under weak selection. Hence, our result can be seen as a connection between the two limit orderings since it involves both, ecological and evolutionary terms in the first order approximation. Thus, our approach does not belong to the class of “first population limit, then selection limit” models as for instance analyzed in Sample and Allen (2017) nor does it belong to the “first weak selection, then large population limit” class but is rather somewhere in between the two limit orderings (even though formally according to Definition 2 provided in Sample and Allen (2017) our limit belongs to the category of $\omega N$-limits). This can also be related to the fact that our approximation holds neither in case $M(1-d/b)\rightarrow \infty$ (typically associated with the $N\omega$-limit) nor for $M(1-d/b)\rightarrow 0$ (associated with the $\omega N$-limit).

Moreover, this sort of breakdown of the $\frac{1}{3}$-rule under demographic fluctuations has also been observed by Ashcroft et al. (2017). Instead, at least in our case, the fixation behavior simply depends on the stability of the internal fixed point of the deterministic system. For a asymptotically stable fixed point the fixation probability of a single mutant is larger than neutral, while for an unstable fixed point it is smaller. This similarity between the general replicator equation, i.e. the deterministic evolutionary behavior in the fixed population size model and the fixation probability holds for both, our stochastically varying population size model, as well as for the model with constant population size derived in Constable and McKane (2015), see also Fig. 7 in Sect. 4.1. Furthermore, the similarity between the fixation probability and its corresponding replicator dynamics is generalizable to models with multiple player interactions under demographic fluctuations (Czuppon and Gokhale 2018). These observations lead us to our conjecture that the qualitative behavior of the fixation probability under weak selection and varying population sizes, at least in a mechanistic modeling approach, is largely influenced by the deterministic replicator dynamics as opposed to the stochastic birth-death dynamics which led to the $\frac{1}{3}$-rule. The main reason for it being the conditions on the fixed point under which ecological and evolutionary processes can be separated.

## Conclusion

The goal of this manuscript is the analysis of the invasion of a mutant strain when introduced into a wild-type population. Assuming a constant population size or deterministically varying population sizes this quantity has already been studied extensively. Here, we extend the analysis to systems including stochastic demographic fluctuations. Dealing with a competitive Lotka–Volterra model we are able to approximate the fixation probability under the weak selection assumption, i.e. the interaction rates between individuals just differ slightly. This mechanistic implementation of the model allows for a straightforward interpretation of the selection effects in terms of competition rates, much in the spirit of Doebeli et al. (2017).

In order to obtain an expression for the fixation probability, we approximate the model by stochastic diffusions and apply tools from stochastic differential equation theory. We observe that the evolutionary success of a mutant mainly depends on its interspecific competition rate *b*. This is due to the resident type being more frequent initially yielding a higher probability for a mutant individual to interact with a resident type. This implies that a larger payoff for the mutant interacting with the wild-type ensures an enhanced fixation probability. This can be seen explicitly by the factor $(1-\frac{d}{b})$ occurring in Eq. (9) which is the only term in the formula that can switch signs given an initially rare mutant, i.e. $p<p^*$.

Still, the values *a* and *c* play a role in the overall evolutionary picture. While lowering *c* increases $p^*$ and thus the region where the invading type has a selective advantage, the parameter *a* has an impact on the overall population size after fixation of the mutant strain. This might end in a decrease of the total number of individuals if $a<d$, even though the mutant has a selective advantage over the wild-type due to $b>d$.

Furthermore, we studied the fixation probability of exactly one mutant in the initial population. Non-surprisingly and as already observed in systems with deterministic population growth/decrease, cf. Kimura and Ohta (1974), we see that the fixation probability monotonically decreases for increasing initial population sizes. Additionally, we find that in our system due to the varying population size the famous $\frac{1}{3}$-rule for fixed population sizes, see Nowak et al. (2004), does not hold anymore. However, we can relate the deterministic internal equilibrium to the intersection of the neutral fixation probability and its counterpart including selection, i.e. $\varphi _{neu}(p^*,z)=\varphi (p^*,z)=p^*$. This has already been observed in Constable and McKane (2015) and Lambert (2006) but not been stated explicitly

We also explored the parameter range under which our modeling assumptions are valid. As it turns out the regime is different when compared to results from population genetics. There, usually $Ms\gg 1$ is assumed while in our framework the approximation holds for $Ms<1$. This is due to the mechanistic implementation of the population dynamics while in population genetics the former condition is usually assumed in order to derive a reasonable stochastic diffusion limit.

The evolutionary result of populations under stochastically fluctuating population sizes has been studied in various scenarios over the last few years (Melbinger et al. 2010; Chotibut and Nelson 2015; Constable et al. 2016; Chotibut and Nelson 2017). The stochasticity of the system as opposed to a deterministic modeling approach allows for different asymptotic behaviors and especially can reverse the deterministic behavior. This triggers the question for calculating fixation probabilities. We added new insight on the impact of the competition parameters on the fixation probability. Furthermore, we showcase a method from stochastic diffusion theory and developed in Lambert (2006) for approximating this quantity at least in the weak selection limit. Even though it is limited to the study of two interacting species, it is adaptable to many other models (and not only Lotka–Volterra-type systems) which include stochastic variation on the population size level.

## Figures and Tables

**Fig. 11:**
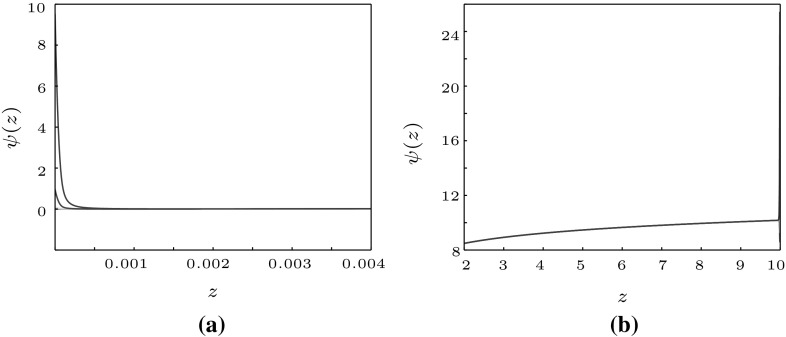
The function $\psi (z)$ is plotted under varying left boundary conditions (**a**) and varying right boundary conditions (**b**)

## Appendix A: Derivation of the diffusion approximation

We derive the Fokker–Planck equation corresponding to our model. The birth- and death-processes are given by

$$\begin{aligned} X \xrightarrow {\beta _X} X + X, \qquad Y\xrightarrow {\beta _Y} Y+Y, \qquad X\xrightarrow {\gamma _X} \varnothing , \qquad Y\xrightarrow {\gamma _Y} \varnothing . \end{aligned}$$

with $\beta _X,\gamma _X$ and $\beta _Y,\gamma _Y$ being the birth and death rates. The competition processes read

$$\begin{aligned} X+X\xrightarrow {\frac{1}{aM}} X,\quad X+Y \xrightarrow {\frac{1}{bM}} Y,\quad X+Y\xrightarrow {\frac{1}{cM}} X,\quad Y+Y \xrightarrow {\frac{1}{dM}} Y, \end{aligned}$$

with *M* scaling the population size in stationarity. We follow the derivation of the Fokker–Planck equation as done in Huang et al. (2015) for the very same model (see also van Kampen 1997; Gardiner 2004). We set

$$\begin{aligned} \begin{aligned} T_X^+&= \beta _x X, \qquad&T_X^-&= \gamma _x X + \frac{1}{aM}X(X-1) + \frac{1}{bM} XY,\\ T_Y^+&= \beta _y Y, \qquad&T_Y^-&= \gamma _y Y + \frac{1}{cM}XY + \frac{1}{dM}Y(Y-1) \end{aligned} \end{aligned}$$

as the transition rates of the system and derive the master equation. That is, for $p_t(X_t,Y_t)$ being the probability for the system to be in state (*X*, *Y*) at time *t* we have

$$\begin{aligned} \begin{aligned} \frac{\partial p_t(X_t,Y_t)}{\partial t}&= T_{X_t-1}^+ p_t(X_t-1,Y_t) + T_{X_t+1}^- p_t(X_t+1,Y_t) \\&\quad +T_{Y_t-1}^+ p_t(X_t,Y_t-1) + T_{Y_t+1}^- p_t(X_t,Y_t+1) \\&\quad - (T_{X_t}^+ +T_{X_t}^- + T_{Y_t}^+ +T_{Y_t}^- ) p_t(X_t,Y_t). \end{aligned} \end{aligned}$$

Rescaling the parameters, i.e. setting $x=\frac{X}{M}$ and $y=\frac{Y}{M}$, we get

$$\begin{aligned} \begin{aligned} \frac{\partial p_t(x_t,y_t)}{\partial t} =&\,T_{X_t-1}^+ p_t(x_t-\tfrac{1}{M},y_t) + T_{X_t+1}^- p_t(x_t+\tfrac{1}{M},y_t) \\&\quad + T_{Y_t-1}^+ p_t(x_t,y_t-\tfrac{1}{M}) + T_{Y_t+1}^- p_t(x_t,y_t+\tfrac{1}{M})\\&\quad - (T_{X_t}^+ + T_{X_t}^- + T_{Y_t}^+ + T_{Y_t}^-) p_t(x_t,y_t) \\ =&\, M \Big [ T_{x_t-\frac{1}{M}}^+ p_t(x_t-\tfrac{1}{M},y_t) + T_{x_t+\frac{1}{M}}^- p_t(x_t+\tfrac{1}{M},y_t) \\&\quad + T_{y_t-\frac{1}{M}}^+ p_T(x_t,y_t-\tfrac{1}{M}) + T_{y_t+\frac{1}{M}}^- p_t(x_t,y_t+\tfrac{1}{M})\\&\quad - (T_{x_t}^+ + T_{x_t}^- + T_{y_t}^+ + T_{y_t}^-) p_t(x_t,y_t)\Big ]. \end{aligned} \end{aligned}$$

In the following we neglect the time subscript of the variables *x* and *y*. Then, doing a Taylor expansion of the function *f* and the transition rates *T* around $(x_t,y_t)$ up to the second order yields

$$\begin{aligned} \frac{\partial p_t(x,y)}{\partial t}= & {} M\Bigg [ \left( T_{x}^+ - \frac{1}{M}\frac{\partial T_x^+}{\partial x} + \frac{1}{2M^2} \frac{\partial ^2 T_x^+}{\partial x^2}\right) \left( p_t(x,y)- \frac{1}{M}\frac{\partial p_t(x,y)}{\partial x} \right. \\&\left. + \frac{1}{2M^2} \frac{\partial ^2 p_t(x,y)}{ \partial x^2}\right) \\&+ \left( T_{x}^- + \frac{1}{M}\frac{\partial T_x^-}{\partial x} + \frac{1}{2M^2}\frac{\partial ^2 T_x^-}{ \partial x^2}\right) \left( p_t(x,y)+ \frac{1}{M}\frac{\partial p_t(x,y)}{\partial x} \right. \\&\left. + \frac{1}{2M^2} \frac{\partial ^2 p_t(x,y)}{ \partial x^2}\right) \\&+ \left( T_{y}^+ - \frac{1}{M} \frac{\partial T_y^+}{\partial y} + \frac{1}{2M^2} \frac{\partial ^2 T_y^+}{ \partial y^2}\right) \left( p_t(x,y)- \frac{1}{M}\frac{\partial p_t(x,y)}{\partial y} \right. \\&\left. + \frac{1}{2M^2} \frac{\partial ^2 p_t(x,y)}{ \partial y^2}\right) \\&+ \left( T_{y}^- + \frac{1}{M} \frac{\partial T_y^-}{\partial y} + \frac{1}{2M^2} \frac{\partial ^2 T_y^-}{ \partial y^2}\right) \left( p_t(x,y)+ \frac{1}{M} \frac{\partial p_t(x,y)}{\partial y} \right. \\&\left. + \frac{1}{2M^2} \frac{\partial ^2 p_t(x,y)}{ \partial y^2}\right) \\&- (T_{x}^+ + T_x^- + T_y^+ + T_y^-) p_t(x,y)\Bigg ]\\= & {} M\Bigg [ - \frac{1}{M} \left( \frac{\partial }{\partial x} \left( T_x^+\ p_t(x,y)\right) \right) + \frac{1}{2M^2}\left( \frac{\partial ^2}{\partial x^2}\left( T_x^+\ p_t(x,y)\right) \right) \\&+ O\left( \frac{1}{M^3}\right) \\&+ \frac{1}{M} \left( \frac{\partial }{\partial x} \left( T_x^-\ p_t(x,y)\right) \right) + \frac{1}{2M^2}\left( \frac{\partial ^2}{\partial x^2}\left( T_x^-\ p_t(x,y)\right) \right) + O\left( \frac{1}{M^3}\right) \\&- \frac{1}{M} \left( \frac{\partial }{\partial y} \left( T_y^+\ p_t(x,y)\right) \right) + \frac{1}{2M^2}\left( \frac{\partial ^2}{\partial y^2}\left( T_y^+\ p_t(x,y)\right) \right) \\&+ O\left( \frac{1}{M^3}\right) \\&+ \frac{1}{N} \left( \frac{\partial }{\partial y} \left( T_y^-\ p_t(x,y)\right) \right) + \frac{1}{2M^2}\left( \frac{\partial ^2}{\partial y^2}\left( T_y^-\ p_t(x,y)\right) \right) \\&+ O\left( \frac{1}{M^3}\right) \Bigg ] \\\approx & {} -\frac{\partial }{\partial x}\left[ (T_x^+ - T_x^-) p_t(x,y)\right] + \frac{1}{2M}\frac{\partial ^2}{\partial x^2}\left[ (T_x^+ + T_x^-) p_t(x,y)\right] \\&-\frac{\partial }{\partial y}\left[ (T_y^+ - T_y^-) p_t(x,y)\right] + \frac{1}{2M}\frac{\partial ^2}{\partial y^2}\left[ (T_y^+ + T_y^-) p_t(x,y)\right] . \end{aligned}$$

Inserting the terms for the transition rates $T_{x/y}^{+/-}$ gives the Fokker–Planck equation which corresponds to the diffusion equation given in Eq. (4), see for example (van Kampen 1997; Gardiner 2004).

## Appendix B: Derivation of the fixation probability

Given the probability density $p_{(x_0,y_0)}(t;x,y)$ which describes the probability of the system given by the equations in (4) to be in state (*x*, *y*) at time *t* if started in $(x_0,y_0)$ the fixation probability is given by

$$\begin{aligned} \varphi (t;x_0,y_0) = \int _0^t p_{(x_0,y_0)}(s;x>0,0)\, ds. \end{aligned}$$

We note, that the condition $(x>0,0)$ in the probability density function ensures that the fixation probability only takes into account the probability mass of trajectories resulting in the extinction of type *Y* individuals under the condition that type *X* individuals are still present.

From Lambert (2005, Theorem 3.5) we know that the population described by the dynamics in Eq. (4) (or more generally a logistic Feller diffusion) goes extinct almost surely for times *t* large enough. Since then $p_{(x_0,y_0)}(t;0,0)\rightarrow 1$ for *t* tending to infinity, the fixation probability $\varphi (x_0,y_0)=\lim _{t\rightarrow \infty } \varphi (t;x_0,y_0)$ satisfies

$$\begin{aligned} \frac{\partial \varphi (t;x_0,y_0)}{\partial t} \rightarrow \frac{\partial \varphi (x_0,y_0)}{\partial t} = 0, \qquad \text { for } t\rightarrow \infty . \end{aligned}$$

On the other hand we have

$$\begin{aligned} \frac{\partial \varphi (x_0,y_0)}{\partial t} = \widetilde{G}\varphi (x_0,y_0), \end{aligned}$$

where the operator $\widetilde{G}$ is given in Eq. (7). Hence, we need to solve $\widetilde{G}\varphi = 0$ with boundary conditions $\varphi (0,y) = 0$ and $\varphi (x,0)=1$ for $x,y>0$ which follow immediately from the model dynamics which then gives the partial differential equation with boundary conditions as stated in Eq. (6).

## Appendix C: Derivation of the transformed generator

The generator of the system of stochastic differential equations given in Eq. (4) reads (for *f* a twice differentiable function)

$$\begin{aligned} \begin{aligned} Gf(x,y)&= x\left( \beta _X-\gamma _X-\frac{x}{a}-\frac{y}{b}\right) \frac{\partial f}{\partial x} + y\left( \beta _Y-\gamma _Y-\frac{x}{c}-\frac{y}{d}\right) \frac{\partial f}{\partial y} \\&\quad +\frac{x}{2 M}\left( \beta _X + \gamma _X +\frac{x}{a}+\frac{y}{b}\right) \frac{\partial ^2 f}{\partial x^2} + \frac{y}{2 M}\left( \beta _Y + \gamma _Y + \frac{x}{c} + \frac{y}{d}\right) \frac{\partial ^2 f}{\partial y^2}. \end{aligned} \end{aligned}$$

For a more general treatment on Markov processes and stochastic differential equations see for instance Kallenberg (2002, Chapter 21). Doing a parameter transformation from the amount of individuals of each type (*x*, *y*) to the fraction of mutants $p=\frac{x}{x+y}$ and the total population size $z=x+y$ we need to translate the derivatives into the new coordinate system. Now we have $x=pz$ and $y=(1-p)z$ which yields

$$\begin{aligned} \begin{aligned} \frac{\partial f}{\partial x}&=\frac{\partial f}{\partial p}\frac{\partial p}{\partial x} + \frac{\partial f}{\partial z}\frac{\partial z}{\partial x} = \frac{1-p}{z}\frac{\partial f}{\partial p} + \frac{\partial f}{\partial z},\\ \frac{\partial f}{\partial y}&=\frac{\partial f}{\partial p}\frac{\partial p}{\partial x} + \frac{\partial f}{\partial z}\frac{\partial z}{\partial x}= -\frac{p}{z}\frac{\partial f}{\partial p} + \frac{\partial f}{\partial z},\\ \frac{\partial ^2 f}{\partial x^2}&= -\frac{2(1-p)}{z^2}\frac{\partial f}{\partial p} + \left( \frac{1-p}{z}\right) ^2 \frac{\partial ^2 f}{\partial p^2} + \frac{2(1-p)}{z}\frac{\partial ^2 f}{\partial p \partial z} + \frac{\partial ^2 f}{\partial z^2},\\ \frac{\partial ^2 f}{\partial y^2}&= \frac{2p}{z^2} \frac{\partial f}{\partial p} + \left( \frac{p}{z}\right) ^2 \frac{\partial ^2 f}{\partial p^2} - \frac{2p}{z}\frac{\partial ^2 f}{\partial p \partial z} + \frac{\partial ^2 f}{\partial z^2}. \end{aligned} \end{aligned}$$

Hence, the generator changes to

$$\begin{aligned} \widetilde{G}f(p,z)&= p(1-p)\left[ \beta _X-\gamma _X -\beta _Y+\gamma _Y -\frac{pz}{a}-\frac{(1-p)z}{b} + \frac{pz}{c} + \frac{(1-p)z}{d}\right. \\&\quad \left. + \frac{1}{zM}\left( -\beta _X-\gamma _X+\beta _Y+\gamma _Y -\frac{pz}{a}-\frac{(1-p)z}{b} + \frac{pz}{c} \right. \right. \\&\quad \left. \left. + \frac{(1-p)z}{d}\right) \right] \frac{\partial f}{\partial p}\\&\quad + z\bigg [p (\beta _X-\gamma _X) + (1-p) (\beta _Y-\gamma _Y) \\&\qquad \qquad \qquad \left. - \frac{p^2 z}{a}-\frac{p(1-p)z}{b} - \frac{p(1-p)z}{c}-\frac{(1-p)^2 z}{d}\right] \frac{\partial f}{\partial z}\\&\quad + \frac{p(1-p)}{2zM}\bigg [(\beta _X+\gamma _X)(1-p) + (\beta _Y+\gamma _Y) p \\&\qquad \qquad \qquad \left. +\frac{p(1-p)z}{a}+\frac{(1-p)^2 z}{b} + \frac{p^2 z}{c} + \frac{p(1-p)z}{d}\right] \frac{\partial ^2 f}{\partial p^2}\\&\quad + \frac{p(1-p)}{M}\left[ \beta _X+\gamma _X - \beta _Y-\gamma _Y + \frac{pz}{a}+\frac{(1-p)z}{b}-\frac{pz}{c}\right. \\&\quad \left. -\frac{(1-p)z}{d}\right] \frac{\partial ^2 f}{\partial p \partial z}\\&\quad + \frac{z}{2M}\bigg [ p (\beta _X+\gamma _X) + (1-p)(\beta _Y+\gamma _Y) \\&\qquad \qquad \qquad \left. +\frac{p^2 z}{a}+ \frac{p(1-p)z}{b} + \frac{p(1-p)z}{c}+ \frac{(1-p)^2 z}{d}\right] \frac{\partial ^2 f}{\partial z^2}. \end{aligned}$$

Setting $\beta _X=\beta _Y=\beta$, $\gamma _X=\gamma _Y=\gamma$ and noting that $(p^*)^{-1} = 1+ \frac{bd}{ac}\frac{(c-a)}{(b-d)}$ we get

$$\begin{aligned} \widetilde{G}f(p,z)&= p(1-p)\left( z+\frac{1}{M}\right) \left( \frac{1}{d}-\frac{1}{b}-p\left( \frac{1}{d}-\frac{1}{b}+\frac{1}{a}-\frac{1}{c}\right) \right) \frac{\partial f}{\partial p}\\&\quad + z\left[ \beta -\gamma -z\left( \frac{1}{d}-p\left( \frac{2}{d}-\frac{1}{c}-\frac{1}{b}\right) \right. \right. \\&\quad \left. \left. +p^2\left( \frac{1}{d}-\frac{1}{b}+\frac{1}{a}-\frac{1}{c}\right) \right) \right] \frac{\partial f}{\partial z}\\&\quad + \frac{p(1-p)}{2zM}\left[ \beta +\gamma + z\left( \frac{1}{b} + p\left( \frac{1}{d}+\frac{1}{a} - \frac{2}{b}\right) \right. \right. \\&\quad \left. \left. -p^2\left( \frac{1}{d}-\frac{1}{b}+\frac{1}{a}-\frac{1}{c}\right) \right) \right] \frac{\partial ^2 f}{\partial p^2}\\&\quad + \frac{p(1-p)z}{M} \left( p\left( \frac{1}{d}-\frac{1}{b}+\frac{1}{a}-\frac{1}{c}\right) - \frac{1}{d}-\frac{1}{b}\right) \frac{\partial ^2 f}{\partial p \partial z}\\&\quad + \frac{z}{2M}\left[ \beta +\gamma + z\left( \frac{1}{d}-p\left( \frac{2}{d}-\frac{1}{c}-\frac{1}{b}\right) \right. \right. \\&\quad \left. \left. +p^2\left( \frac{1}{d}-\frac{1}{b}+\frac{1}{a}-\frac{1}{c}\right) \right) \right] \frac{\partial ^2 f}{\partial z^2} \\&= p(1-p)\left( \frac{1}{d}-\frac{1}{b}\right) \left( z+\frac{1}{M}\right) \left( 1-p\left( 1+\frac{\frac{1}{a}-\frac{1}{c}}{\frac{1}{d}-\frac{1}{b}}\right) \right) \frac{\partial f}{\partial p}\\&\quad + z\left[ \beta -\gamma -\frac{z}{d}\left( 1-p\left( 2-\frac{d}{c}-\frac{d}{b}\right) \right. \right. \\&\quad \left. \left. +p^2\left( 1-\frac{d}{b}\right) \left( 1+\frac{\frac{1}{a}-\frac{1}{c}}{\frac{1}{d}-\frac{1}{b}}\right) \right) \right] \frac{\partial f}{\partial z}\\&\quad + \frac{p(1-p)}{2zM}\left[ \beta +\gamma + \frac{z}{d}\left( \frac{d}{b} + p\left( 1+\frac{d}{a} - 2\frac{d}{b}\right) \right. \right. \\&\quad \left. \left. -\,p^2\left( 1-\frac{d}{b}\right) \left( 1+\frac{\frac{1}{a}-\frac{1}{c}}{\frac{1}{d}-\frac{1}{b}}\right) \right) \right] \frac{\partial ^2 f}{\partial p^2}\\&\quad + \frac{p(1-p)z}{d M} \left( 1-\frac{d}{b}\right) \left( p\left( 1+\frac{\frac{1}{a}-\frac{1}{c}}{\frac{1}{d}-\frac{1}{b}}\right) - 1\right) \frac{\partial ^2 f}{\partial p \partial z}\\&\quad + \frac{z}{2M}\left[ \beta +\gamma + \frac{z}{d}\left( 1-p\left( 2-\frac{d}{c}-\frac{d}{b}\right) \right. \right. \\&\quad \left. \left. +\,p^2\left( 1-\frac{d}{b}\right) \left( 1+\frac{\frac{1}{a}-\frac{1}{c}}{\frac{1}{d}-\frac{1}{b}}\right) \right) \right] \frac{\partial ^2 f}{\partial z^2} \\&=p(1-p)\frac{1}{d}\left( 1-\frac{d}{b}\right) \left( z+\frac{1}{M}\right) \left( 1-\frac{p}{p^*}\right) \frac{\partial f}{\partial p}\\&\quad + z\left[ \beta -\gamma -\frac{z}{d}\left( 1-p\left( 2-\frac{d}{c}-\frac{d}{b}\right) +\left( 1-\frac{d}{b}\right) \frac{p^2}{p^*}\right) \right] \frac{\partial f}{\partial z}\\&\quad + \frac{p(1-p)}{2zM}\left[ \beta +\gamma + \frac{z}{d}\left( \frac{d}{b} + p\left( 1+\frac{d}{a} - 2\frac{d}{b}\right) \right. \right. \\&\quad \left. \left. -\, \left( 1-\frac{d}{b}\right) \frac{p^2}{p^*}\right) \right] \frac{\partial ^2 f}{\partial p^2}\\&\quad + \frac{p(1-p)z}{d M} \left( 1-\frac{d}{b}\right) \left( \frac{p}{p^*} - 1\right) \frac{\partial ^2 f}{\partial p \partial z}\\&\quad + \frac{z}{2M}\left[ \beta +\gamma + \frac{z}{d} \left( 1 - p\left( 2- \frac{d}{c} -\frac{d}{b}\right) + \left( 1-\frac{d}{b}\right) \frac{p^2}{p^*} \right) \right] \frac{\partial ^2 f}{\partial z^2}, \end{aligned}$$

which is precisely Eq. (7).

## Appendix D: Proof of Theorem 1

Both, Theorems 1 and 2, can be proved in a similar way. Due to this we will only give the Proof of Theorem 1.

First, we recall the conditions needed for the Theorem:

(i) $$(1-\frac{d}{b})^2\ll 1$$,
(ii) $$(1-\frac{d}{b})(2-\frac{d}{c}-\frac{d}{b}) \ll 1$$,
(iii) $$(1-\frac{d}{b})(1+\frac{d}{a}-2\frac{d}{b}) \ll 1$$ and
(iv) $$(1-\frac{d}{b})(\frac{1}{p^*}-2) \ll 1$$.

The Theorem we want to prove is:

### Theorem 1

(Fixation probability under weak selection) Under conditions (i)–(iv) the solution of Eq. (8) can be written as

9 $$\begin{aligned} \varphi (p,z) = p + p(1-p)\left( 1-\frac{p}{p^*}\right) \left( 1-\frac{d}{b}\right) \psi (z) + O\left( \left( 1-\frac{d}{b}\right) ^2\right) , \end{aligned}$$

where $\psi (z)$ is independent of the initial frequency of mutants *p* and solves

10 $$\begin{aligned} \begin{aligned} 0&= \left( z+\frac{1}{M}\right) + z((\beta -\gamma )d-z) \psi '(z) - \frac{3}{zM}\left( (\beta +\gamma )d+z\right) \psi (z)\\&\quad + \frac{z}{2M}((\beta +\gamma )d+z) \psi ''(z). \end{aligned} \end{aligned}$$

### Proof

In order to determine the equation for $\psi (z)$ we apply the generator $\widetilde{G}$ to $\varphi (p,z)$ in Eq. (5), which gives:

$$\begin{aligned} \widetilde{G}\varphi (p,z)= & {} \frac{p(1-p)}{d}\left( 1-\frac{p}{p^*}\right) \left( 1-\frac{d}{b}\right) \left( z+\frac{1}{M}\right) \left( 1+O\left( 1-\frac{d}{b}\right) \right) \\&+ z\left[ \beta -\gamma -\frac{z}{d}\left( 1-p\left( 2-\frac{d}{c}-\frac{d}{b}\right) +\left( 1-\frac{d}{b}\right) \frac{p^2}{p^*}\right) \right] \\&\times p(1-p)\left( 1-\frac{p}{p^*}\right) \left( 1-\frac{d}{b}\right) \psi '(z)\\&+ \frac{p(1-p)}{2zM}\left[ \beta +\gamma + \frac{z}{d}\left( \frac{d}{b} + p\left( 1+\frac{d}{a} - 2\frac{d}{b}\right) - \left( 1-\frac{d}{b}\right) \frac{p}{p^*}\right) \right] \\&\times \left[ \frac{6p}{p^*} - 2 \left( 1 + \frac{1}{p^*} \right) \right] \left( 1-\frac{d}{b}\right) \psi (z)\\&+ \frac{p(1-p)z}{d M} \left( 1-\frac{d}{b}\right) \left( \frac{p}{p^*} - 1\right) O\left( 1-\frac{d}{b}\right) \\&+ \frac{z}{2M}\left[ \beta +\gamma + \frac{z}{d} \left( 1 - p\left( 2- \frac{d}{c} -\frac{d}{b}\right) + \left( 1-\frac{d}{b}\right) \frac{p}{p^*} \right) \right] \\&\times p(1-p)\left( 1-\frac{p}{p^*}\right) \left( 1-\frac{d}{b}\right) \psi ''(z). \end{aligned}$$

Next, applying the weak selection limit, i.e. using conditions (i)–(iii) we obtain

21 $$\begin{aligned} \begin{aligned} \widetilde{G}\varphi (p,z)&\approx \frac{p(1-p)}{d}\left( 1-\frac{p}{p^*}\right) \left( 1-\frac{d}{b}\right) \left( z+\frac{1}{M}\right) \\&\quad + z\left[ \beta -\gamma -\frac{z}{d}\right] p(1-p)\left( 1-\frac{p}{p^*}\right) \left( 1-\frac{d}{b}\right) \psi '(z)\\&\quad + \frac{p(1-p)}{2zM}\left[ \beta +\gamma + \frac{z}{b}\right] \left[ \frac{6p}{p^*}-2 \left( 1 +\frac{1}{p^*}\right) \right] \left( 1-\frac{d}{b}\right) \psi (z) \\&\quad + \frac{z}{2M}\left[ \beta +\gamma + \frac{z}{d}\right] p(1-p)\left( 1-\frac{p}{p^*}\right) \left( 1-\frac{d}{b}\right) \psi ''(z). \end{aligned} \end{aligned}$$

To simplify the term before $\psi (z)$, we observe that

$$\begin{aligned} \begin{aligned} \beta +\gamma + \frac{z}{b} = \beta +\gamma + \frac{z}{d}-\frac{z}{d} \left( 1- \frac{d}{b} \right) = \beta +\gamma + \frac{z}{d}+O \left( 1- \frac{d}{b} \right) \end{aligned} \end{aligned}$$

and additionally that

$$\begin{aligned} 1+\frac{1}{p^*} = 1 + 2 + \frac{1}{p^*}-2=3 + \frac{1}{p^*}-2 \end{aligned}$$

hold. This yields

$$\begin{aligned} \frac{6p}{p^*}-2 \left( 1 +\frac{1}{p^*}\right) = 6\left( \frac{p}{p^*}-1\right) -2\left( \frac{1}{p^*}-2\right) . \end{aligned}$$

Next, we insert these approximations into Eq. (21) and apply condition (iv) such that the last term from above vanishes. Finally, setting $\widetilde{G}\varphi =0$ and dividing by $\frac{p(1-p)}{d}(1-\frac{p}{p^*})(1-\frac{d}{b})$ gives

$$\begin{aligned} \begin{aligned} 0&= \left( z+\frac{1}{M}\right) + z((\beta -\gamma )d-z)\psi '(z) - \frac{3}{zM}\left( (\beta +\gamma )d+z\right) \psi (z) \\&\quad + \frac{z}{2M}((\beta +\gamma )d+z) \psi ''(z). \end{aligned} \end{aligned}$$

This gives Eq. (10) and finishes the proof. $\square$

## Appendix E: Proof of Lemma 1

In this section, we prove that the solution $\psi (z)$ of Eq. (10), i.e.

$$\begin{aligned} \begin{aligned} 0&= \left( z+\frac{1}{M}\right) + z((\beta -\gamma )d-z)\psi '(z) - \frac{3}{zM}\left( (\beta +\gamma )d+z\right) \psi (z) \\&\quad + \frac{z}{2M}((\beta +\gamma )d+z) \psi ''(z). \end{aligned} \end{aligned}$$

is positive. Since the following section is very technical the reader who is satisfied with a numerical argument should skip this section and continue reading at “Appendix F”.

### Remark 6

The proof goes along the same lines as that of a similar result derived in Lambert (2006, Theorem 3.5).

Before we prove Lemma 1 we recall and actually rewrite Eq. (10). We know that $\psi (z)$ is the solution to the following ordinary differential equation:

22 $$\begin{aligned} \begin{aligned} v(z)&= \left( z+\frac{1}{M}\right) \\&= - z((\beta -\gamma )d-z) \psi '(z) + \frac{3}{zM}\left( (\beta +\gamma )d+z\right) \psi (z) \\&\quad - \frac{z}{2M}((\beta +\gamma )d+z) \psi ''(z). \end{aligned} \end{aligned}$$

The first step is to rewrite Eq. (22). It is a Riccati-type equation and it is convenient for these to do the following transformation: $h(z)=-\frac{\psi '(z)}{\psi (z)}$. This yields

$$\begin{aligned} h'(z) = -\frac{\psi ''(z)}{\psi (z)} + h^2 (z) \end{aligned}$$

and hence when considering the homogeneous differential Eq. (22), i.e. setting $v(z)=0$, we obtain

$$\begin{aligned} z((\beta -\gamma )d -z)h + \frac{3}{zM}\left( (\beta +\gamma )d + z\right) +\frac{z}{2M}((\beta +\gamma )d + z)(h'(z)-h^2(z)) = 0, \end{aligned}$$

Rearranging terms, this equation reads

23 $$\begin{aligned} h'(z)-h^2(z) + 2M\frac{(\beta -\gamma )d -z}{(\beta +\gamma )d + z}h(z) + \frac{6}{z^2}=0. \end{aligned}$$

Now, the proof of Lemma 1 consist of the following two steps:

(i) Solve Eq. (23) and show that the solution is integrable in $[0,\infty )$. (Lemma 3)
(ii) Construct the solution of Eq. (22) using the homogeneous solution characterized in Lemma 3.

Before we prove these two steps we state an auxiliary lemma, which we will make frequent use of.

### Lemma 2

Let *f*(*t*) be a real function with constant sign and rational behavior at $+\infty$. Then for $z\rightarrow \infty$ we have

$$\begin{aligned} \begin{aligned} \chi (z)&=6e^{2M(z-2d\beta \ln ((\beta +\gamma )d+z)} \int _z^\infty f(t) e^{-2M(t -2d\beta \ln ((\beta +\gamma )d+t))} dt\\&= 6 e^{m(z)}\int _z^\infty f(t)e^{-m(t)}dt \sim \frac{3f(z)}{M}. \end{aligned} \end{aligned}$$

### Proof

The result follows by partial integration. See also Lemma A.1 in Lambert (2006). $\square$

We start with the first step.

### Lemma 3

Equation (23) has a unique and non-negative solution *h* which satisfies

- $$\limsup _{z\rightarrow \infty } z^2 h(z)\le \frac{3}{M},$$
- for $z\rightarrow 0+$ we find $h(z)= \frac{2}{z} + \frac{M(\beta -\gamma )}{\beta +\gamma } + O(z)$.

### Remark 7

Note, that the two statements characterizing the limit behavior of *h* can be read off by forming the corresponding limits in Eq. (23).

### Proof

In order to show that *h* is unique and non-negative, we set

$$\begin{aligned} \xi (z) = \int _0^z e^{m(x)}dx, \end{aligned}$$

with $m(x):= 2M(x-2d\beta \ln ((\beta +\gamma )d + x))$. Note that $\xi :[0,\infty ) \rightarrow [0,\infty )$ is increasing and a bijection which allows us to define $\eta :=\xi ^{-1}$. It holds $\eta '(x) = \exp (-m(\eta (x)))>0$. Furthermore, the derivative of *m* satisfies

$$\begin{aligned} m'(x) = -2M\frac{(\beta -\gamma )d-x}{(\beta +\gamma )d+x}. \end{aligned}$$

Next, let *w* solve

24 $$\begin{aligned} w' - w^2 = -6\left( \frac{\eta '}{\eta }\right) ^2. \end{aligned}$$

Then, $h(z)=e^{m(z)}w(\xi (z))$ solves Eq. (23) which can be seen by the following calculation:

$$\begin{aligned} \begin{aligned} h'(z)-h^2(z)&= h(z)m'(z) + e^{m(z)} w'(\xi (z))\xi '(z) - e^{2m(z)}w^2(\xi (z)) \\&= h(z)m'(z) - e^{2m(z)} 6 \left( \frac{\eta '(\xi (z))}{\eta (\xi (z))}\right) ^2 \\&= h(z)m'(z) - \frac{6}{z^2}. \end{aligned} \end{aligned}$$

Hence, *h* solves Eq. (23) if and only if *w* solves Eq. (24). Redoing the proofs of Lemma 4.1, Lemma 4.2(i) and Lemma 4.3 of Lambert (2005) in our setting (which follow step-by-step in the same way and are therefore spared out) and arguing in the same vein as in the proof of Lemma 2.1 from Lambert (2005) (again step-by-step) we obtain that *w* is the unique non-negative solution to Eq. (24). Furthermore, these results imply the following properties

(i) $$w\rightarrow 0$$ for $$z\rightarrow \infty $$,
(ii) $$w\rightarrow \infty $$ for $$z\rightarrow 0$$,
(iii) *w* decreases for *z* tending to $\infty$,
(iv) *w* decreases in the neighborhood of $0+$,
(v) $w\le \sqrt{6}\frac{\eta '}{\eta }$ in the neighborhood of $0+$ and $\infty$.

Due to the definition of *h* we can deduce that it is unique and non-negative and satisfies

(i) $$h\rightarrow \infty $$ for $$z\rightarrow 0+$$,
(ii) $$0<h(z)<\frac{\sqrt{6}}{z}$$.

Next we examine the limit behavior of *h*. Mimicking the proof of Lemma 3.4 in Lambert (2006) we show

$$\begin{aligned} \limsup _{z\rightarrow \infty } h(z) z^2 \le \frac{1}{2M}. \end{aligned}$$

In order to prove this, we set

$$\begin{aligned} \chi (z)=6\exp \left( m(z)\right) \int _z^\infty x^{-2} \exp \left( -m(x))\right) dx, \end{aligned}$$

with

$$\begin{aligned} \chi '(z) = m'(z)\chi (z)-\frac{6}{z^2} = -2M\frac{(\beta -\gamma )d-z}{(\beta +\gamma )d +z} \chi (z) -\frac{6}{z^2}. \end{aligned}$$

This yields

$$\begin{aligned} \begin{aligned} (h-\chi )'(z)&= h(z)m'(z) + e^{2m(z)} w'(\xi (z)) - m'(z)\chi (z) + \frac{6}{z^2}\\&= h(z)m'(z) - m'(z)\chi (z) + e^{2m(z)} w^2(\xi (z))\\&> m'(z)(h(z)-\chi (z)). \end{aligned} \end{aligned}$$

For *z* large enough, we have that $m'(z)>0$. Hence, whenever $h(z)>\chi (z)$ this gives $(h-\chi )'(z)>0$ which means that from that point on $h>\chi$. By Gronwall-type reasoning (see Lambert 2006, Lemma 3.4) and for the Lemma of Ethier and Kurtz 1986, Theorem A.5.1) we see that then *h* tends to infinity. However, this is a contradiction to $h(z)\le \frac{\sqrt{6}}{z}$. Thus, $h<\chi$ and therefore with Lemma 2 we have $\limsup _{z\rightarrow \infty } h(z)z^2 \le \frac{3}{M}$. This shows statement (a).

Next we turn our attention to the limit behavior of *h* when *z* approaches 0 from above. Therefore, instead of $z>0$ we consider $c:=-z$ which simplifies the following reasoning. We define

$$\begin{aligned} \zeta (c):= -\frac{c h(-c) + 2}{c^3} e^{-m(-c)}. \end{aligned}$$

Noting that

$$\begin{aligned} h'(-c) +\frac{6}{c^2} - h(-c) m'(-c) = h^2(-c) \end{aligned}$$

we get

$$\begin{aligned} \begin{aligned} \zeta '(c)&= -\frac{(h(-c)-c h'(-c))c^3 - 3c^2 (c h(-c)+2)}{c^6}e^{-m(-c)} \\&\quad -\frac{c h(-c)+2}{c^3}m'(-c)e^{-m(-c))} \\&= -\frac{1}{c^3}\left( -2h(-c) -c\left( h'(-c)+\frac{6}{c^2}-m'(-c)h(-c)\right) + 2m'(-c)\right) e^{-m(-c)}\\&= -\frac{1}{c^3}\left( -h(-c)(2+ch(-c))\right) e^{-m(-c)}-\frac{2}{c^3}e^{-m(-c)} \\&=-h(-c)\zeta (c) - \frac{2}{c^3}m'(-c) e^{-m(-c))}\\&=-h(-c)\zeta (c) + \frac{4M}{c^3}\frac{(\beta -\gamma )d+c}{(\beta +\gamma )d -c}e^{-m(-c)}. \end{aligned} \end{aligned}$$

For $c<0$ close enough to 0 we see that $\zeta$ has constant sign in the neighborhood of $0-$ since if for some $c_0$ we have $\zeta (c_0)=0$ then $\zeta '(c_0)<0$. Thus, we have either (1) $\zeta >0$ or (2) $\zeta <0$ in the neighborhood of $0-$. This yields in case (1)

$$\begin{aligned} |\zeta (c)|' = \zeta '(c) < \frac{2}{|c|^3}|m'(-c)| e^{-m(-c)} \end{aligned}$$

and in case (2)

$$\begin{aligned} |\zeta (c)|' = -\zeta '(c) = h(-c)\zeta (c) + \frac{2}{c^3} m'(-c)e^{-m(-c)} < \frac{2}{|c^3|}|m'(-c)| e^{-m(-c)}. \end{aligned}$$

Thus, in both cases $|c^3| |\zeta (c)|'$ is strictly bounded from above near $0-$ which yields that $|\zeta (c)|\in O(\frac{1}{c^2})$. But this means that $h(z)\sim \frac{2}{z}$ for $z\rightarrow 0+$.

For the second order term we consider the auxiliary function

$$\begin{aligned} \tau (z) := \frac{h(z)-\frac{2}{z}}{z^4}. \end{aligned}$$

Again, we calculate the derivative and obtain

$$\begin{aligned} \tau '(z)= & {} \frac{(h'(z)+\frac{2}{z^2})z^4 -4z^3 h(z)+8z^2}{z^8}\\= & {} \frac{1}{z^4}\left( h'(z)-h^2(z) + h^2(z) + \frac{2}{z^2} -4\frac{h(z)}{z} + \frac{8}{z^2}\right) \\= & {} \frac{1}{z^4}\left( h(z)m'(z) -\frac{6}{z^2} + \frac{10}{z^2} + h^2(z) - 4\frac{h(z)}{z}\right) \\= & {} \frac{1}{z^4}\left( \left( h(z)-\frac{2}{z}\right) ^2 + m'(z)\left( h(z)-\frac{2}{z}\right) + m'(z)\frac{2}{z}\right) \\= & {} \frac{1}{z^4}\left( h(z)-\frac{2}{z}\right) ^2 + \frac{m'(z)}{z^4}\left( h(z)-\frac{2}{z}\right) \\&+\frac{1}{z^4}\left( \frac{4M}{(\beta +\gamma )d + z} - \frac{4M(\beta -\gamma )d}{z((\beta +\gamma )d+z)}\right) . \end{aligned}$$

Multiplying with $z^4$ we see that the first three terms on the right side are bounded for $z\rightarrow 0+$ whereas the last term is of order $z^{-1}$. This yields

$$\begin{aligned} \tau (z) \approx \frac{1}{z^4}\frac{M(\beta -\gamma )}{(\beta +\gamma )} + O\left( \frac{1}{z^3}\right) \qquad \text {for } z\rightarrow 0+. \end{aligned}$$

This finishes the proof of statement (b), i.e. $h(z) = \frac{2}{z} + \frac{M(\beta -\gamma )}{\beta +\gamma } + O(z)$ for $z\rightarrow 0+$. $\square$

Based on these estimates of the homogeneous solution *h* we can now continue by solving the inhomogeneous Eq. (10), see also Eq. (22). Our goal now is to prove Lemma 1 from the main text which states that the function $\psi (z)$ solving this equation is positive.

### Proof of Lemma 1

Again, we follow the reasoning of the corresponding proof given in Lambert (2006).

First of all note that due to Lemma 3 (a) and (b) *h* is integrable at $\infty$ and that as $z\rightarrow 0+$ we find

25 $$\begin{aligned} \int _z^\infty h(t)dt = \int _z^1 h(t)-\frac{2}{t}dt + \int _z^1 \frac{2}{t}dt + \int _1^\infty h(t) dt = \alpha + \ln (z^{-2}) + O(z), \end{aligned}$$

where $\alpha$ is given by

$$\begin{aligned} \alpha := \int _z^1 h(t)-\frac{2}{t}dt + \int _1^\infty h(t) dt. \end{aligned}$$

Therefore we can define

26 $$\begin{aligned} B(z):= \psi (z) \exp \left( -\int _z^\infty h(t) dt\right) , \end{aligned}$$

where $\psi$ is the solution of Eq. (22) and h solves Eq. (23). In the following we will prove a representation of $B(z) = B(0) + \int _0^z B'(x)dx$ which will then give an explicit expression for $\psi (z)$. This expression will then show that $\psi$ is indeed positive for all *z*.

So, let us start by analyzing *B*(*z*). Differentiating *B*(*z*) gives

$$\begin{aligned} \begin{aligned} B'(z)&= \left( \psi '(z) + \psi (z)h(z)\right) \exp \left( -\int _z^\infty h(t) dt\right) ,\\ B''(z)&= \left( \psi ''(z) + 2\psi '(z)h(z)+ \psi (z)h'(z) + \psi (z)h^2(z) \right) \exp \left( -\int _z^\infty h(t) dt\right) . \end{aligned} \end{aligned}$$

Next, we look for a combination of these derivatives such that these give the inhomogeneous solution $v(z) = z+\frac{1}{M}$. This will then allow us to write down an explicit expression of $B'(z)$ which can then be translated to an explicit expression of $\psi$. Using that $\psi$ and *h* are solutions of the differential equations given in (22) and (23), respectively, we calculate

27 $$\begin{aligned}&\frac{1}{6}B''(z) - \frac{1}{3}h(z)B'(z) + \frac{M}{3}\frac{(\beta -\gamma )d-z}{(\beta +\gamma )d+z}B'(z)\nonumber \\&\quad = \frac{1}{6} \left( \psi ''(z) + 2\psi '(z)h(z)+ \psi (z)h'(z) + \psi (z)h^2(z) \right) e^{-\int _z^\infty h(t) dt} \nonumber \\&\quad \quad - \frac{1}{3}h(z)\left( \psi '(z) + \psi (z)h(z)\right) e^{-\int _z^\infty h(t) dt} \nonumber \\&\quad \quad + \frac{M}{3}\frac{(\beta -\gamma )d-z}{(\beta +\gamma )d+z}\left( \psi '(z) + \psi (z)h(z)\right) e^{-\int _z^\infty h(t) dt}\nonumber \\&\quad = \left( \frac{1}{6}\psi ''(z) + \frac{M}{3}\frac{(\beta -\gamma )d-z}{(\beta +\gamma )d+z}\psi '(z) + \frac{1}{3}\psi '(z)h(z)-\frac{1}{3}\psi '(z)h(z)\right. \nonumber \\&\quad \quad + \psi (z)\underbrace{\left( \frac{1}{6}(h'(z)+h^2(z))-\frac{1}{3}h^2(z) + \frac{M}{3}\frac{(\beta -\gamma )d-z}{(\beta +\gamma )d+z} h(z)\right) }_{=-\frac{1}{z^2} \text { cf. } (23)}\Bigg )e^{-\int _z^\infty h(t)dt}\nonumber \\&\quad = \left( \frac{1}{6}\psi ''(z) + \frac{M}{3}\frac{(\beta -\gamma )d-z}{(\beta +\gamma )d+z} \psi '(z) -\frac{1}{z^2}\psi (z)\right) e^{-\int _z^\infty h(t)dt}\nonumber \\&\quad {\mathop {=}\limits ^{(22)}} -v(z)\frac{M}{3z((\beta +\gamma )d+z)}e^{-\int _z^\infty h(t)dt}. \end{aligned}$$

In order to determine *B*(*z*) we make use of another auxiliary function

28 $$\begin{aligned} C(z):= \frac{1}{6}B'(z)\exp \left( 2\int _z^\infty h(t) dt - m(z)\right) . \end{aligned}$$

Again we calculate the derivative and, this time applying Eq. (27), we obtain

$$\begin{aligned}\begin{aligned} C'(z)&= \left( \frac{1}{6}B''(z) -\frac{1}{3}h(z)B'(z) + \frac{M}{3}\frac{(\beta -\gamma )d-z}{(\beta +\gamma )d+z} B'(z)\right) e^{\int _z^\infty h(t)dt - m(z)} \\&=-\frac{M v(z)}{3z((\beta +\gamma )d+z)}e^{\int _z^\infty h(t)dt - m(z)}. \end{aligned} \end{aligned}$$

Integrating from *z* to $\infty$ gives

$$\begin{aligned} \begin{aligned} C(z) = C(\infty ) + \int _z^\infty \frac{M v(t)}{3t((\beta +\gamma )d+t)}e^{\int _t^\infty h(s)ds - m(t)}dt, \end{aligned} \end{aligned}$$

which with Eq. (28) yields

29 $$\begin{aligned} B'(z)= & {} 6 C(\infty ) e^{-2\int _z^\infty h(t) dt + m(z)} \nonumber \\&\qquad + 6 e^{-2\int _z^\infty h(t) dt + m(z)} \int _z^\infty \frac{M v(t)}{3t((\beta +\gamma )d+t)}e^{\int _t^\infty h(s)ds -m(t)}dt.\nonumber \\ \end{aligned}$$

Applying Lemma 2 we see that the second term behaves like

$$\begin{aligned} \frac{v(z)}{z((\beta +\gamma )d+z)}=\frac{z+\frac{1}{M}}{z((\beta +\gamma )d+z)}, \text { as } z\rightarrow \infty , \end{aligned}$$

which implies that

$$\begin{aligned} B'(z)\sim 6 B(\infty ) e^{m(z)}, \end{aligned}$$

as $z\rightarrow \infty$. Due to Eq. (26) $\psi$ increases with *z* faster than exponential which we show is not true. Therefore, $C(\infty )$ needs to be zero giving an explicit expression for $B'$.

Hence, let us consider the model for very large values of *z*. This implies that the system is governed by the quadratic competition terms and can be approximated by the corresponding ODE-system which reads:

$$\begin{aligned}\begin{aligned} \dot{x}_t&= - \frac{x_t^2}{a}-\frac{x_t y_t}{b},\\ \dot{y}_t&= - \frac{x_t y_t}{c} -\frac{y_t^2}{d}. \end{aligned} \end{aligned}$$

The dynamics of the fraction of mutants, i.e. *p*, is then given by

$$\begin{aligned} \dot{p}_t= & {} \frac{dx_t(x_t+y_t) -x_t(dx_t +dy_t)}{(x_t+y_t)^2} \\= & {} -\frac{p^2 z}{a} - \frac{p(1-p)z}{b}\\&+ \frac{pz}{z^2} \left( \frac{p^2z^2}{a} + \frac{p(1-p)z^2}{b}+\frac{p(1-p)z^2}{c}+\frac{(1-p)^2 z^2}{d}\right) \\= & {} zp(1-p)\left( \frac{(1-p)}{d}+\frac{p}{c}-\frac{(1-p)}{b}-\frac{p}{a}\right) \\= & {} zp(1-p)\left( \frac{1}{d}-\frac{1}{b}\right) \left( 1-p\left( 1+\frac{\frac{1}{a}-\frac{1}{c}}{\frac{1}{d}-\frac{1}{b}}\right) \right) \\= & {} \frac{zp(1-p)}{d}\left( 1-\frac{d}{b}\right) \left( 1-\frac{p}{p^*}\right) . \end{aligned}$$

From heuristic reasoning it is clear that for large *z* the fixation probability $\varphi$ does not change under slight variation of *p* and *z*, i.e. $\varDelta \varphi =\varphi (p_{t+\delta t},z_{t+\delta t})-\varphi (p_t,z_t)$ vanishes with $z_{t+\delta t}=z_t-\delta z$ and

$$\begin{aligned} \begin{aligned} p_{t+\delta t}&= p_t+\frac{z_t p_t(1-p_t)}{d}\left( 1-\frac{d}{b}\right) \left( 1-\frac{p_t}{p^*}\right) \delta t\\&= p_t+\frac{z_t p_t(1-p_t)}{d}\left( 1-\frac{d}{b}\right) \left( 1-\frac{p_t}{p^*}\right) \\&\quad \delta t\frac{\dot{z}_t}{z_t^2\left( -\frac{p_t^2}{a}-\frac{p_t(1-p_t)}{b}-\frac{p_t(1-p_t)}{c}-\frac{(1-p_t)^2}{d}\right) }\\&= p_t+\frac{z_t p_t(1-p_t)}{d}\left( 1-\frac{d}{b}\right) \left( 1-\frac{p_t}{p^*}\right) \\&\quad \delta t \frac{\dot{z}_t}{-\frac{z_t^2}{d}\left( 1-p_t\left( 2-\frac{d}{c}-\frac{d}{b}\right) +\left( 1-\frac{d}{b}\right) \frac{p_t^2}{p^*}\right) }. \end{aligned} \end{aligned}$$

Forming the formal derivative and noting that $\frac{\partial \varphi }{\partial p}\approx 1$ due to the weak selection assumption we have

$$\begin{aligned} 0 \approx \frac{d\varphi }{dt} =\frac{\partial \varphi }{\partial p}\frac{dp}{dt} + \frac{\partial \varphi }{\partial z} \frac{dz}{dt} = \frac{(p_{t+\delta t}-p_t)}{\delta t} + \frac{\partial \varphi }{\partial z}\dot{z}. \end{aligned}$$

This gives

$$\begin{aligned} \frac{\partial \varphi }{\partial z}\sim \frac{p(1-p)\left( 1-\frac{d}{b}\right) \left( 1-\frac{p}{p^*}\right) }{z\left( 1-p\left( 2-\frac{d}{c}-\frac{d}{b}\right) +\left( 1-\frac{d}{b}\right) \frac{p^2}{p^*}\right) }, \quad \text {for } z\rightarrow \infty \end{aligned}$$

and therefore $\psi '(z)\sim \frac{1}{z}$ for $z\rightarrow \infty$ under weak selection. This shows that

30 $$\begin{aligned} \psi (z)\sim \ln (z) \text { as } z\rightarrow \infty . \end{aligned}$$

Applying this and Lemma 3 (a) to Eq. (26) we find for $z\rightarrow \infty$ that

$$\begin{aligned} \begin{aligned} B'(z) = (\psi '(z) + \psi (z)h(z)) e^{-\int _z^\infty h(t)dt} \sim \left( \frac{1}{z} +\frac{\ln (z)}{z^2}\right) e^{\frac{1}{z}} \rightarrow 0, \end{aligned} \end{aligned}$$

which due to Eq. (28) implies that $C(\infty )$ needs to be 0 in order to provide the right limit behavior of *B*(*z*).

Lastly, we investigate the limit behavior of *B* as *z* tends to 0. Here, applying Eq. (25) we find that Eq. (29) for $z\rightarrow 0+$ satisfies

$$\begin{aligned} B'(z) \sim e^{-2\ln (z^{-2})} 2 \int _z^\infty \frac{Mt+1}{t((\beta +\gamma )d+t)}e^{\ln (t^{-2})} dt \sim z^4 \int _z^\infty \frac{2}{t^3} dt = z^2. \end{aligned}$$

Thus, *B* is integrable at $0+$ giving

$$\begin{aligned} \int _0^z B'(t)dt = B(z)-B(0) = \psi (z)\exp \left( -\int _z^\infty h(t)dt\right) -B(0). \end{aligned}$$

The left hand side vanishes for $z\rightarrow 0+$ which with Eq. (25) implies that

$$\begin{aligned} \psi (z) \sim B(0)\exp \left( \int _z^\infty h(t)dt\right) =\frac{B(0)e^{\alpha }}{z^2}. \end{aligned}$$

If *B*(0) were not 0 this would mean that $\psi (z)$ is unbounded for $z\rightarrow 0+$. But, for very small populations individuals do not sense any density dependence which translates to the competition processes not affecting the fixation probability. Hence, the governing equations read

$$\begin{aligned} \begin{aligned} dx_t&= (\beta -\gamma )x + \frac{1}{\sqrt{M}}\sqrt{(\beta +\gamma )x}dW^1\\ dy_t&= (\beta -\gamma )y + \frac{1}{\sqrt{M}}\sqrt{(\beta +\gamma )y}dW^2. \end{aligned} \end{aligned}$$

This system is equivalent to a neutral model, i.e. the fixation probability is independent of the initial population size. Hence, $\psi$ needs to approach 0 for small *z* which indeed shows $B(0)=0$.

This result and Eq. (26) allow us to write

31 $$\begin{aligned} \begin{aligned} \psi (z)&= e^{\int _z^\infty h(t)dt} \int _0^z e^{-2\int _t^\infty h(s)ds+ m(t)} \int _t^\infty \frac{2M + \frac{2}{t}}{(\beta +\gamma )d + t} e^{\int _s^\infty h(u)du -m(t)} ds dt. \end{aligned} \end{aligned}$$

Thus, we find an explicit form of $\psi$ which indeed shows that it is a non-negative function. This finishes the proof. $\square$

### Remark 8

The positivity of $\psi$ solving Eq. (15) in the case of a dominance game can be shown in a similar way.

## Appendix F: Numerical evaluation of $\psi (z)$

In order to calculate values of $\psi$ we solve Eq. (10) numerically. For this we use the predefined function “solve_bvp” from the scipy.integrate library in Python, Jones et al. (2001). Therefore we need to input boundary values for the algorithm to work with. In particular we evaluate $\psi$ in the interval [0.01, 10] with boundary values $\psi (0.01) = \frac{0.01}{3(\beta +\gamma )d}$ and $\psi (10)=\ln (10)$. The justification for choosing $\psi (10)=\ln (10)$ is based on Eq. (30) above. On the other hand, for very small values of *z* we use the following reasoning.

We need to examine the behavior of Eq. (31) for $z\rightarrow 0+$. Therefore, we consider the following notation:

$$\begin{aligned} \psi (z) = e^{\int _z^\infty h(t)dt} \int _0^z e^{-2\int _t^\infty h(s)ds}H(t) dt, \end{aligned}$$

with

$$\begin{aligned} H(z) = e^{-2M(2d\beta \ln ((\beta +\gamma )d + z)-z)}\int _z^\infty f(t) e^{2M(2d\beta \ln ((\beta +\gamma )d+t)-t)} dt, \end{aligned}$$

where

$$\begin{aligned} f(z) = \left( \frac{2M + \frac{2}{z}}{(\beta +\gamma )d + z}\right) e^{\int _t^\infty h(t)dt}. \end{aligned}$$

Next, using Eq. (25) we have for $z\rightarrow 0+$ that

$$\begin{aligned} f(z) \sim \left( \frac{2M + \frac{2}{z}}{(\beta +\gamma )d + z}\right) \frac{1}{z^2} e^{\alpha } \sim \frac{2}{z^3(\beta +\gamma )d} e^{\alpha } \end{aligned}$$

and therefore

$$\begin{aligned} \begin{aligned} H(z)&\sim e^{-2M(2d\beta \ln ((\beta +\gamma )d + z)-z)}\int _z^\infty \frac{2}{z^3(\beta +\gamma )d} e^{\alpha } e^{2M(2d\beta \ln ((\beta +\gamma )d+t)-t)} dt\\&\sim \frac{1}{z^2(\beta +\gamma )d}e^{\alpha }. \end{aligned} \end{aligned}$$

Inserting this into $\psi$ we first get

$$\begin{aligned} \begin{aligned} \int _0^z e^{-2\int _t^\infty h(s)ds}H(t) dt&\sim \int _0^z e^{-2\alpha } t^4 \frac{1}{t^2(\beta +\gamma )d}e^{\alpha }dt\\&= \int _0^z e^{-\alpha } \frac{t^2}{(\beta +\gamma )d} dt\\&= \frac{z^3}{3(\beta +\gamma )d} e^{-\alpha }, \end{aligned} \end{aligned}$$

which yields

$$\begin{aligned} \psi (z) \sim e^{\int _z^\infty h(t)dt} \frac{z^3}{3(\beta +\gamma )d} e^{-\alpha } = z^{-2}e^{\alpha }\frac{z^3}{3(\beta +\gamma )d}e^{-\alpha } = \frac{z}{3(\beta +\gamma )d}. \end{aligned}$$

This shows the limit behavior of $\psi$ for $z\rightarrow 0+$. Note, that the limit behavior for $z\rightarrow 0+$ can also be read off Eq. (10) by only considering the leading terms in *z*.

Finally, we show in Fig. 11 how the function $\psi$ depends on different choices of boundary values. In subfigure (a) we varied the initial values at $z=0.0001$ from $10^{-8}$ to 10 while setting $\psi (10)=\ln (10)$. We see that it only affects the values very close to the initial point of the numerical implementation. The same holds if we vary the boundary values at $z=10$, i.e. $\psi (10)$ goes from 1 to 100. Holding the initial value $\psi (0.0001)=\frac{0.0001}{3(\beta +\gamma )d}$ fixed we only see variation close to the right boundary.

## Appendix G: Derivation of Eq. (20)

In this section we apply the methods derived in Constable and McKane (2015) and later generalized in Constable and McKane (2017) to our model. We will closely follow the reasoning in Constable and McKane (2015).

The strategy is as follows: we first identify a neutral manifold which corresponds to the stable population size under neutral evolution of the types. Next, we derive a one-dimensional stochastic diffusion along this manifold and see that under neutral parameter choices the process is purely stochastic. Introducing a weak selection into the system leads then to a stochastic differential equation with both a deterministic part coming from the selection structure of the model and a stochastic term emerging from the neutral component. We will now follow these steps to derive the fixation probability under deterministic population size changes.

The first step is to identify the center manifold. Equation 9 in the reference provides a closed form for it. Translated to our setting we get

$$\begin{aligned} z = x_1 + x_2 = a (\beta -\gamma )=:q, \end{aligned}$$

where *a* is the inverse competition coefficient in the neutral scenario.

The next step is to derive a one-dimensional stochastic diffusion which can then be used to calculate fixation probabilities. The idea is that under neutral model assumptions there is no deterministic drift along the central manifold and all the dynamics can be reduced to a stochastic diffusion term. The variable along the manifold is given by $q=\frac{z}{a(\beta -\gamma )}$ and is restricted to the interval [0, 1]. The stochastic diffusion is then given by (see Eqs. (14) and (15) in Constable and McKane 2015)

$$\begin{aligned} dq = \sqrt{B(q)}dW, \quad \text {with} \quad B(q) = \frac{2\beta a}{M(\beta -\gamma )^2} q(1-q). \end{aligned}$$

Lastly, we need to identify the deterministic part of the system with weak selection. Therefore, we need to compute Eq. 19 in the reference. In order to do so, we need to transform their variables into our parameter framework. Transforming the birth- and death rates is straightforward while for competition rates we need to do some arithmetics. The competition coefficients in Constable and McKane (2015), denoted by $c_{ij}$, can be written as follows, exemplarily done for $c_{12}$:

$$\begin{aligned} \frac{1}{b} = c_{12} = c_0(1+\varepsilon \gamma _{12}) \quad \Leftrightarrow \quad \gamma _{12} = \frac{1}{\varepsilon }\left( \frac{1}{b c_0}-1\right) . \end{aligned}$$

As the reference case we set $c_0=1$ such that we can now transform between the two systems. Their Eq. 19 now transforms to

$$\begin{aligned} \begin{aligned} A(q)&\approx \varepsilon q(1-q)[(\gamma _{11}-\gamma _{12}) - (\gamma _{11}+\gamma _{22} - \gamma _{12}-\gamma _{21}) q] \\&= q(1-q)\left[ \frac{1}{d}-\frac{1}{b} - \left( \frac{1}{a}+\frac{1}{d} -\frac{1}{b} -\frac{1}{c}\right) q\right] . \end{aligned} \end{aligned}$$

In summation we now have expressions for *B*(*q*) and *A*(*q*) which not yield the stochastic differential equation

$$\begin{aligned} dq = A(q)dt + \sqrt{B(q)}dW_t, \end{aligned}$$

which is also stated in Eq. (20) in the main text.
